# Heat-inactivated modified vaccinia virus Ankara boosts Th1 cellular and humoral immunity as a vaccine adjuvant

**DOI:** 10.1038/s41541-022-00542-5

**Published:** 2022-10-19

**Authors:** Ning Yang, Aitor Garcia, Cindy Meyer, Thomas Tuschl, Taha Merghoub, Jedd D. Wolchok, Liang Deng

**Affiliations:** 1 Dermatology Service, Department of Medicine, Memorial Sloan Kettering Cancer Center, New York, NY 10065 USA; 2 Laboratory of RNA Molecular Biology, The Rockefeller University, New York, NY 10065 USA; 3 Immuno-oncology service, Human Oncology and Pathogenesis Program, Memorial Sloan Kettering Cancer Center, New York, NY 10065 USA; 4 Parker Institute for Cancer Immunotherapy, Memorial Sloan Kettering Cancer Center, New York, NY USA; 5 Department of Medicine, Weill Cornell Medicine, New York, NY USA; 6 Department of Pharmacology, Weill Cornell Medicine, New York, NY USA; 7 Sandra and Edward Meyer Cancer Center, Weill Cornell Medicine, New York, NY USA; 8 Weill Cornell Medical College, New York, NY USA

**Keywords:** Adjuvants, Pox virus

## Abstract

Protein or peptide-based subunit vaccines have generated excitement and renewed interest in combating human cancer or COVID-19 outbreak. One major concern for subunit vaccine application is the weak immune responses induced by protein or peptides. Developing novel and effective vaccine adjuvants are critical for the success of subunit vaccines. Here we explored the potential of heat-inactivated MVA (heat-iMVA) as a vaccine adjuvant. Heat-iMVA dramatically enhances T cell responses and antibodies responses, mainly toward Th1 immune responses when combined with protein or peptide-based immunogen. The adjuvant effect of Heat-iMVA is stronger than live MVA and is dependent on the cGAS/STING-mediated cytosolic DNA-sensing pathway. In a therapeutic vaccination model based on tumor neoantigen peptide vaccine, Heat-iMVA significantly extended the survival and delayed tumor growth. When combined with SARS-CoV-2 spike protein, Heat-iMVA induced more robust spike-specific antibody production and more potent neutralization antibodies. Our results support that Heat-iMVA can be developed as a safe and potent vaccine adjuvant for subunit vaccines against cancer or SARS-CoV-2.

## Introduction

Vaccination is one of the most efficient strategies to prevent infectious diseases, to control a pandemic such as COVID-19, and to avert or eliminate cancers associated with a viral infection such as human papillomavirus (HPV)-associated cervical cancer. Vaccination with live vaccinia virus (VACV) or the highly attenuated modified vaccinia virus Ankara (MVA) generated robust and durable immune responses, which led to smallpox eradication officially announced by WHO in 1980 during the 33rd World Health Assembly^1^, and possible persistent protection in previously vaccinated individuals^2^. Although the mechanisms of live attenuated vaccine (LAV)-mediated protective immune responses are not entirely understood, it is accepted that innate immune-sensing mechanisms of dendritic cells activated by LAVs play an essential role in shaping adaptive immunity^3^.

In addition to LAVs and viral vector- or nucleic acid-based vaccines, subunit vaccination based on recombinant protein or peptides as antigens is another widely used vaccination platform. These include licensed vaccines against influenza virus, hepatitis B virus (HBV), HPV, varicella-zoster (VZV), and many other pathogens^4^. Furthermore, discoveries of cancer neoantigens generated by somatic mutations in cancer cells, have brought excitement and renewed interest in cancer vaccines^5–9^ and personalized neoantigen peptide vaccination has shown promising results in clinical trials^6,10,11^.

Because recombinant protein or peptide vaccines usually generate weak immune responses, safe and effective vaccine adjuvants that boost vaccine efficacy are urgently needed. Licensed vaccine adjuvants include inorganic aluminum salts (alum), the oil-in-water emulsion MF59, monophosphoryl lipid A (MPL) absorbed on aluminum salts (AS04), and the toll-like receptor 9 (TLR9) agonist CpG 1018^4^. In addition to TLR agonists, agents that activate the cytosolic pattern recognition receptors, for example, stimulator of interferon genes (STING) agonists, have also been explored as vaccine adjuvants^12,13^. It has been postulated that vaccine adjuvants that mimic natural infection such as LAV might elicit potent and durable immune responses via the activation of innate immune-sensing pathways^14^.

VACV belongs to the poxvirus family, and modified vaccinia virus Ankara (MVA) is a highly attenuated vaccinia strain derived from chorioallantois vaccinia virus Ankara (CVA) through more than 500 passages in chicken embryo fibroblasts^15,16^. MVA is a safe and effective vaccine against smallpox and monkeypox and a viral vector against other infectious agents^16–24^. We and others have previously shown that VACV infection of bone marrow-derived dendritic cells (BMDCs) fails to induce type I IFN production. By contrast, MVA infection induces IFN production via the cGAS/STING-mediated cytosolic DNA-sensing pathway^25,26^.

VACV encodes many immunomodulatory genes to evade the host immune system^27–29^. Inactivation of VACV or MVA, by heating VACV or MVA at 55 °C for 1 h, reduces infectivity by more than 1000-fold and much more potently induces type I IFN production than live viruses in conventional DCs (cDCs) or plasmacytoid DCs (pDCs)^30–32^. The paramunity-inducing effect of MVA or inactivated MVA was demonstrated before^33–36^, suggesting MVA or inactivated MVA has the potential used as vaccine adjuvant. Based on its safety and immune-stimulating features, we hypothesized that the heat-inactivated VACV or MVA could act as vaccine adjuvant because: (i) it mimics natural infection except for lost replication capacity; (ii) it induces type I IFN production in cDCs via the cGAS/STING pathway and in pDCs via the TLR9/TLR7/MyD88 pathway^31,32^; (iii) immunosuppressive genes antagonizing type I IFN production/signaling encoded by the MVA genome were not expressed in heat-inactivated MVA (heat-iMVA) infected cells. Here, we show that heat-iMVAm is a stronger vaccine adjuvant than live MVA and it can boost the T and B cell responses of subunit vaccines. Furthermore, co-administration of heat-iMVA with tumor neoantigen peptides delays tumor growth and prolongs mouse survival in a syngeneic B16-F10 melanoma model. Co-delivery of heat-iMVA with SARS-CoV-2 spike protein enhances the production of spike-specific neutralizing antibodies. In summary, our results provide proof-of-concept for heat-iMVA as a vaccine adjuvant against infectious diseases and cancers.

## Results

### Co-administration of chicken ovalbumin with heat-iMVA enhances the generation of ovalbumin-specific cellular and humoral immune responses in mice

We have previously shown that compared with live MVA, heat-inactivation of MVA results in stronger induction of type I IFN and proinflammatory cytokines and chemokines via the activation of the cyclic GMP-AMP synthase/stimulator of interferon gene (cGAS/STING) pathway in DCs^25,31^. In addition, intratumoral (IT) delivery of heat-iMVA generates stronger antitumor immunity than live MVA in immune-competent murine tumor models^25,31^.

**Fig. 1:**
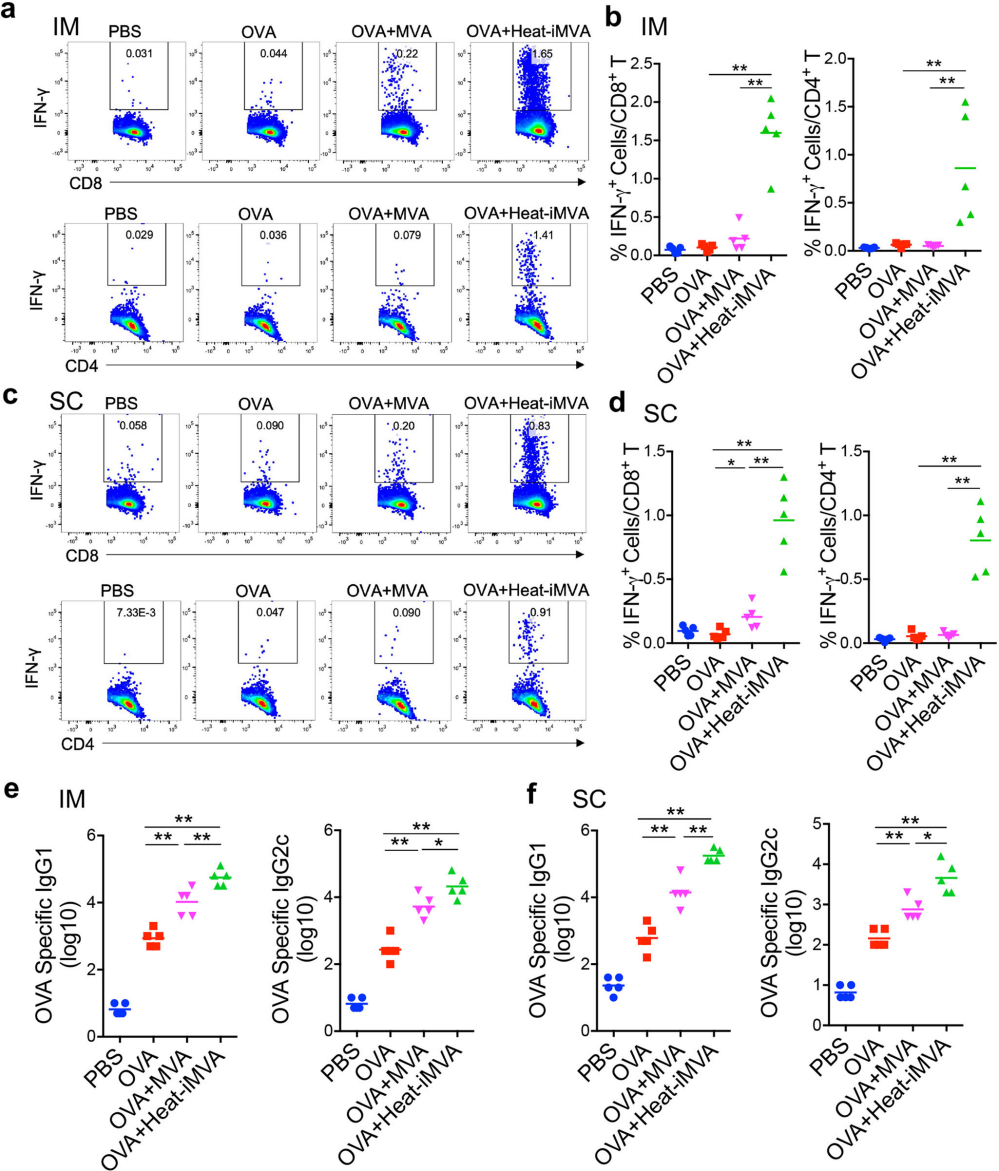
Co-administration of heat-inactivated MVA (heat-iMVA) enhances antigen-specific T cell and antibody responses after intramuscular (IM) vaccination with chicken ovalbumin (OVA). WT C57BL/6J mice were vaccinated on day 0 and day 14 with OVA (10 μg), OVA (10 μg) plus MVA (10^7^ pfu) or OVA (10 μg) plus heat-iMVA (an equivalent amount of 10^7^ pfu/mouse) intramuscularly (IM) (**a**, **b**, **e**) or subcutaneously (SC) (**c**, **d**, **f**). On day 21, splenocytes (**a**–**d**) were stimulated with OVA_257-264_ (CD8^+^ T specific peptide) or OVA_323-339_ peptide (CD4^+^ T specific peptide). The expression of IFN-γ by CD8^+^ T cells or CD4^+^ T was measured by flow cytometry. **e**, **f** OVA-specific immunoglobulin G1 (IgG1) or OVA-specific immunoglobulin G2c (IgG2c) titers in the serum from PBS, OVA, OVA + MVA, or OVA + heat-iMVA-vaccinated mice were determined by ELISA. Data are represented as mean ± SEM (*n* = 3–5; ^*^P < 0.05 and ^**^P < 0.01; Two-tailed Mann–Whitney *U* test). Data are representative of two independent experiments.

Here, we hypothesized that heat-iMVA could be used as a vaccine adjuvant to boost antigen-specific cellular and humoral immune responses in vivo. To test this hypothesis, we first prime-immunized mice intramuscularly (IM) or subcutaneously (SC) with the model antigen ovalbumin (OVA; 10 µg) with or without MVA (10^7^ pfu) or heat-iMVA (an equivalent of 10^7^ pfu), followed by a boost-immunization two weeks later, and euthanized them one week after the boost vaccination, with spleens and blood subsequently collected and assessed for OVA-specific T-cells and antibodies. To determine anti-OVA CD8^+^ T-cell responses, we incubated splenocytes with OVA_257__–__264_ peptide, an MHC class I (K^b^)-restricted peptide epitope of OVA for 12 h, followed by staining with anti-CD8 and anti-IFN-γ antibodies. To test anti-OVA CD4^+^ T-cell responses, we incubated splenocytes with OVA_323-339_ peptide, an MHC class II I-A^d^-restricted peptide epitope of OVA for 12 h, followed by staining with anti-CD4 and anti-IFN-γ antibodies. IM administration of OVA plus heat-iMVA increased splenic anti-OVA IFN- γ ^+^CD8^+^ T-cells and anti-OVA IFN- γ ^+^CD4^+^ T-cells compared with OVA alone, while OVA plus live MVA had minimal effect on the generation of OVA-specific T cells in the spleens. The percentages of IFN-γ^+^ T-cells among splenic CD8^+^ T-cells increased from 0.1% in the OVA-treated mice to 1.6% in OVA + heat-iMVA-treated ones (*P* < 0.01; *n* = 5; Fig. 1a, b). Moreover, the percentages of IFN-γ^+^ T-cells among splenic CD4^+^ T-cells increased from 0.064% in the OVA-treated mice to 0.86% in OVA + Heat-iMVA-treated mice (*P* < 0.05; *n* = 5; Fig. 1a, b). Although the combination of OVA plus live MVA modestly enhanced the production of OVA-specific IgG1 and IgG2c compared with OVA alone, OVA plus heat-iMVA induced stronger IgG1 and IgG2c production than OVA alone or OVA plus live MVA (Fig. 1e). IgG2c antibody titers were upregulated by 100-fold in the OVA plus heat-iMVA group than OVA alone (*P* < 0.001; *n* = 5; Fig. 1e), suggesting that prime-boost vaccination with OVA plus heat-iMVA induced stronger Th1 immune responses.

Subcutaneous (SC) vaccination with OVA plus heat-iMVA resulted in more potent OVA-specific CD8^+^ or CD4^+^ T cell responses and higher titers of OVA-specific IgG1 and IgG2c than OVA alone or OVA plus live MVA (Fig. 1c, d, f). Together, these results indicate that heat-iMVA can be used as a vaccine adjuvant to enhance both cellular and humoral Th1-biased immune responses.

### Heat-iMVA promotes more robust Th1 responses and IgG2c production compared with complete Freund adjuvant (CFA) and AddaVax

**Fig. 2:**
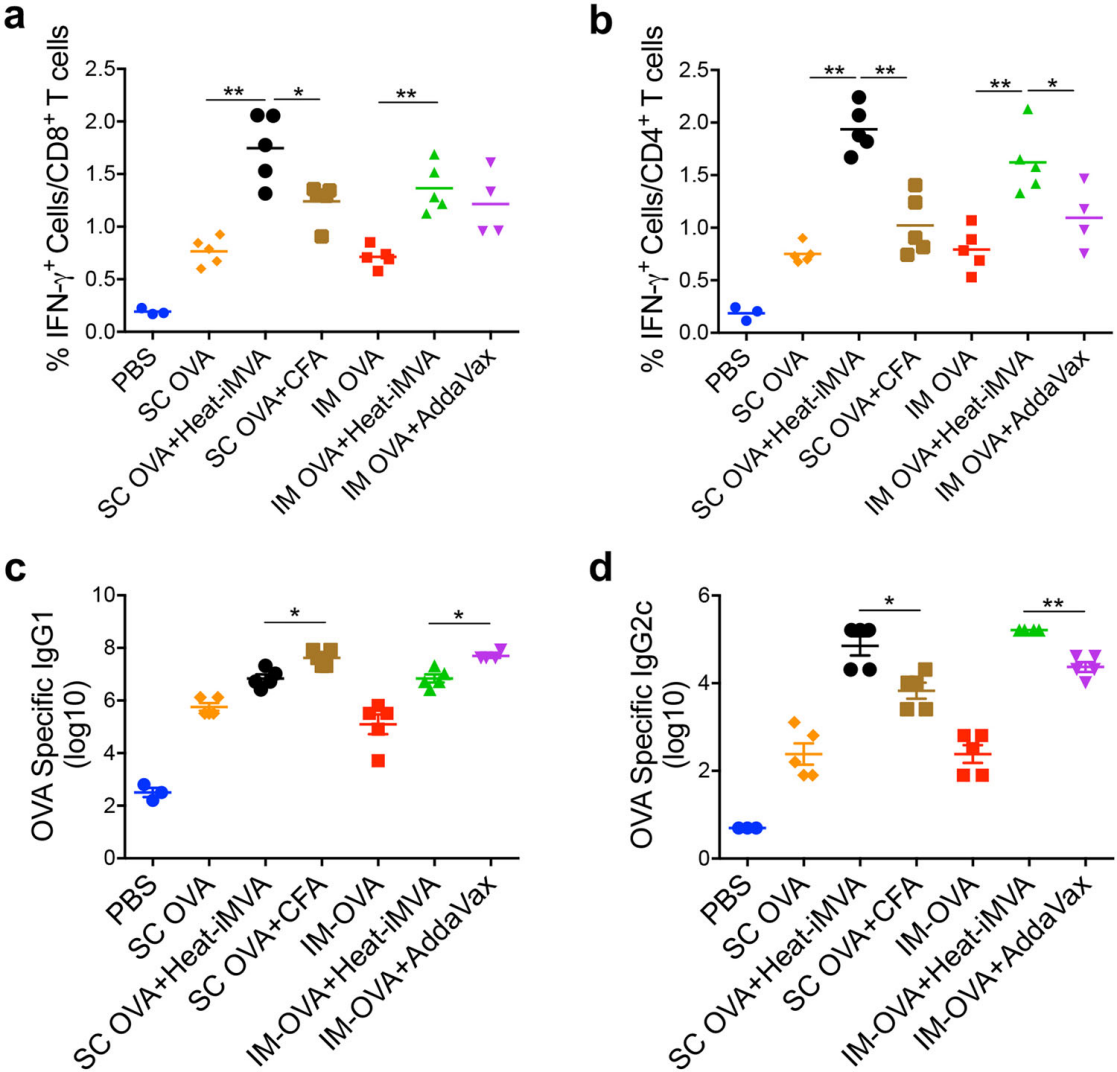
Heat-iMVA promotes stronger antigen-specific Th1 responses and IgG2c production compared with complete Freund adjuvant (CFA) and AddaVax after cutaneous vaccination. Antigen-specific T cell and antibodies responses were measured after intramuscular (IM) or subcutaneous (SC) vaccination on day 0 and day 14 with OVA (10 μg) in the presence or absence of heat-iMVA (an equivalent amount of 10^7^ pfu) in C57BL/6J mice. **a**, **b** On day 21, splenocytes were stimulated with OVA_257__–__264_ or OVA_323__–__339_. The expression of IFN-γ by CD8^+^ or CD4^+^ T cells was measured by flow cytometry. **c**, **d** On day 21, OVA-specific immunoglobulin G1 (IgG1) or OVA-specific immunoglobulin G2c (IgG2c) titers in the were determined by ELISA. Data are represented as mean ± SEM (*n* = 3–5; ^*^P < 0.05 and ^**^P < 0.01; Two-tailed Mann–Whitney *U* test). Data are representative of two independent experiments.

Next, we compared the adjuvanticity of heat-iMVA with other well-known vaccine adjuvants. For example, CFA comprises heat-killed *Mycobacterium tuberculosis* in non-metabolizable oils (paraffin oil and mannide monooleate) and also contains ligands for TLR2, TLR4, and TLR9. Injection of antigen with CFA induces a Th1-dominant immune response^37^. Although CFA’s use in humans is currently impermissible due to its toxicity profile, it is commonly used in animal studies because of its strong adjuvant effects. To test whether heat-iMVA is superior to CFA, we prime-vaccinated mice SC with OVA plus heat-iMVA or OVA plus CFA followed by a boost-vaccination two weeks later. Subsequently, we harvested spleens, dLNs, and blood one week after the boost-vaccination to analyze anti-OVA CD8^+^ and CD4^+^ T-cell and antibody responses. SC administration of OVA plus heat-iMVA induced higher levels of antigen-specific CD8^+^ and CD4^+^ T-cells than OVA plus CFA in the spleens of vaccinated mice. The percentage of IFN-γ^+^ T-cells among CD8^+^ T-cells in the spleens increased from 0.77% in the OVA-treated mice to 1.7% in OVA + heat-iMVA-treated mice as opposed to 1.2% in OVA + CFA-treated mice (*P* < 0.05; *n* = 5; OVA + Heat-iMVA vs. OVA + CFA; Fig. 2a). The percentage of IFN-γ^+^ T-cells among CD4^+^ T-cells in the spleens increased from 0.75% in the OVA-treated mice to 1.9% in OVA + heat-iMVA-treated mice as opposed to 1.0% in OVA + CFA group (*P* < 0.001; *n* = 5; OVA + heat-iMVA vs. OVA + CFA; Fig. 2b). We also observed that serum IgG1 titers from OVA + CFA-immunized mice were 6-fold higher than those in the serum from OVA + heat-iMVA-immunized mice (*P* < 0.01; *n* = 5; OVA + Heat-iMVA vs. OVA + CFA; Fig. 2c), whereas serum IgG2c titers from OVA + CFA-immunized mice were 10-fold lower than those in the serum of OVA + heat-iMVA-immunized mice (*P* < 0.01; *n* = 5; OVA + Heat-iMVA vs. OVA + CFA; Fig. 2d). IgG1 is considered a “Th2-like” isotype, whereas IgG2c is considered a “Th1-like” isotype. These results indicate that co-administration of OVA plus Heat-iMVA promotes stronger Th1-biased humoral immunity than OVA plus CFA.

MF59, a squalene-based oil-in-water vaccine adjuvant in the inactivated influenza vaccine Fluad (licensed for use in adults aged 65 and older), is also the adjuvant in subunit vaccines against SARS-CoV-2^38^. AddaVax is an MF59-like preclinical grade nano-emulsion that induces both Th1-cellular immune responses and Th2-biased humoral responses^39^. Here, we observed that intramuscular (IM) vaccination with OVA plus heat-iMVA induced CD8^+^ T cell responses similar to OVA plus AddaVax, however, the former combination promoted higher CD4^+^ T cell responses than the latter (Fig. 2a, b). The percentage of IFN-γ^+^ T-cells among splenic CD4^+^ T-cells increased from 0.79% in the OVA-treated mice to 1.6% in OVA + heat-iMVA-treated mice as opposed to 1.1% in OVA + AddaVax group (*P* < 0.05; *n* = 5; OVA + heat-iMVA vs. OVA + AddaVax; Fig. 2b). In addition, IM vaccination of OVA plus heat-iMVA induced 7-fold higher OVA-specific IgG2c titers (*P* < 0.01; *n* = 5; OVA + heat-iMVA vs. OVA + AddaVax; Fig. 2d) and 7-fold lower OVA-specific IgG1 than OVA plus AddaVax (*P* < 0.001; *n* = 5; OVA + heat-iMVA vs. OVA + AddaVax; Fig. 2c), suggesting that co-administration of the antigen plus heat-iMVA more potently induces antigen-specific Th1-biased cellular and humoral immune responses compared with combining the antigen with AddaVax. Overall, SC or IM co-administration of OVA with heat-iMVA generated similar cellular and humoral immune responses to OVA (Fig. 2a–d).

### Heat-iMVA-induced vaccine adjuvant effects depend on CD103^+^ /CD8α^+^ DCs and the STING pathway

**Fig. 3:**
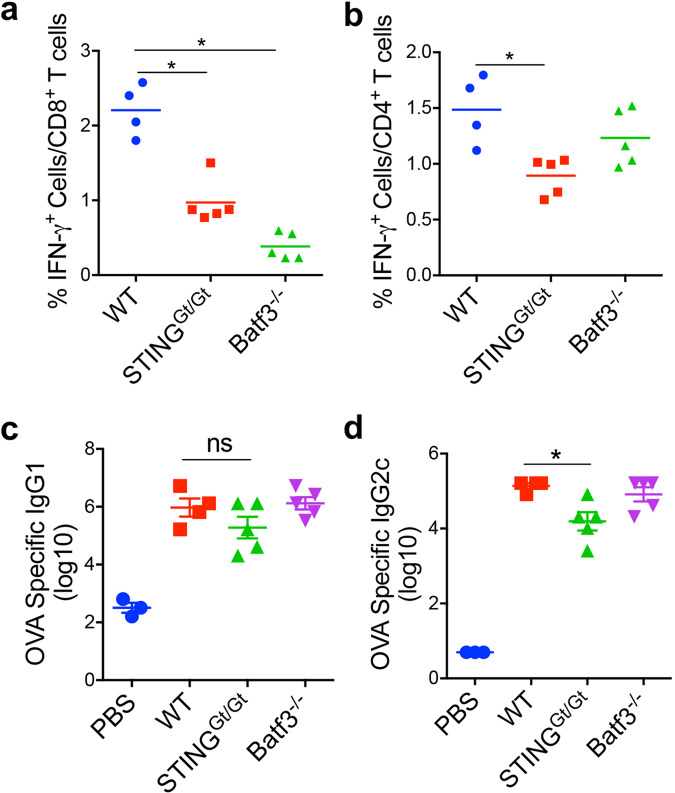
CD103^+^ DC and the cGAS/STING pathway contribute to heat-iMVA adjuvanticity. STING^Gt/Gt^, Batf3^−/−^, or age-matched WT C57BL/6J mice were intramuscularly vaccinated on day 0 and day 14 with OVA (10 µg) + heat-iMVA (an equivalent of 10^7^ pfu). **a**, **b** On day 21, mice were euthanized, and spleens and blood were collected. splenocytes were stimulated with OVA_257__–__264_ or OVA_323__–__339_. The expression of IFN-γ by CD8^+^ or CD4^+^ T cells was measured by flow cytometry. **c**, **d** On day 21, OVA-specific immunoglobulin G1 (IgG1) or OVA-specific immunoglobulin G2c (IgG2c) titers in the serum were determined by ELISA. Data are represented as mean ± SEM (*n* = 3–5; ^*^P < 0.05; Two-tailed Mann–Whitney *U* test). Data are representative of three independent experiments.

BATF3 is a transcription factor critical for the development of CD103^+^/CD8α^+^ lineage DCs, which plays an essential role in cross-presenting viral and tumor antigens^40^. To test whether STING or Batf3 plays a role in heat-iMVA-mediated vaccine adjuvant effects, we SC vaccinated age-matched WT C57B/6, STING^Gt/Gt^, or Batf3^−/−^ mice with OVA + heat-iMVA twice, two weeks apart. Our results showed that the percentages of anti-OVA IFN-γ^+^ T-cells among splenic CD8^+^ T-cells induced by heat-iMVA were reduced from 2.2% in WT mice to 0.38% in Batf3^−/−^ mice (*P* < 0.001; *n* = 5; WT vs. Batf3^−/−^; Fig. 3a), whereas the generation of splenic anti-OVA IFN-γ^+^ CD4^+^ T-cells seemed unaffected (Fig. 3b), with minimal effects on the IgG1 and IgG2c production (Fig. 3c, d). These results support a role for Batf3-dependent CD103^+^/CD8α^+^ DCs in cross-presenting OVA antigen to generate OVA-specific splenic CD8^+^ T-cells in our vaccination model.

STING agonist cGAMP can be used as a vaccine adjuvant^41^. Here, we observed that the percentage of anti-OVA IFN-γ^+^ T-cells among splenic CD8^+^ T-cells induced by heat-iMVA decreased from 2.2% in WT mice to 0.97% in STING^Gt/Gt^ mice (*P* < 0.01; *n* = 5; WT vs. STING^Gt/Gt^; Fig. 3a). STING-deficiency also reduced the percentage of splenic anti-OVA IFN-γ^+^ CD4^+^ T-cells among total splenic CD4^+^ T-cells from 1.5% in WT mice to 0.9% in STING^Gt/Gt^ mice (*P* < 0.01; *n* = 5; WT vs. STING^Gt/Gt^; Fig. 3b). Moreover, serum IgG2c titers were reduced by 10-fold in the STING^Gt/Gt^ mice vaccinated with OVA + heat-iMVA compared with immunized WT mice (*P* < 0.05; *n* = 5; WT vs. STING^Gt/Gt^; Fig. 3d), while serum IgG1 titers did not significantly differ between the two groups (Fig. 3c). These results demonstrate that the cGAS/STING-mediated cytosolic DNA-sensing pathway plays a critical role in the vaccine adjuvant effects of heat-iMVA.

### Heat-iMVA enhances antigen cross-presentation by BMDCs and proliferation of antigen-specific T cells in vitro

**Fig. 4:**
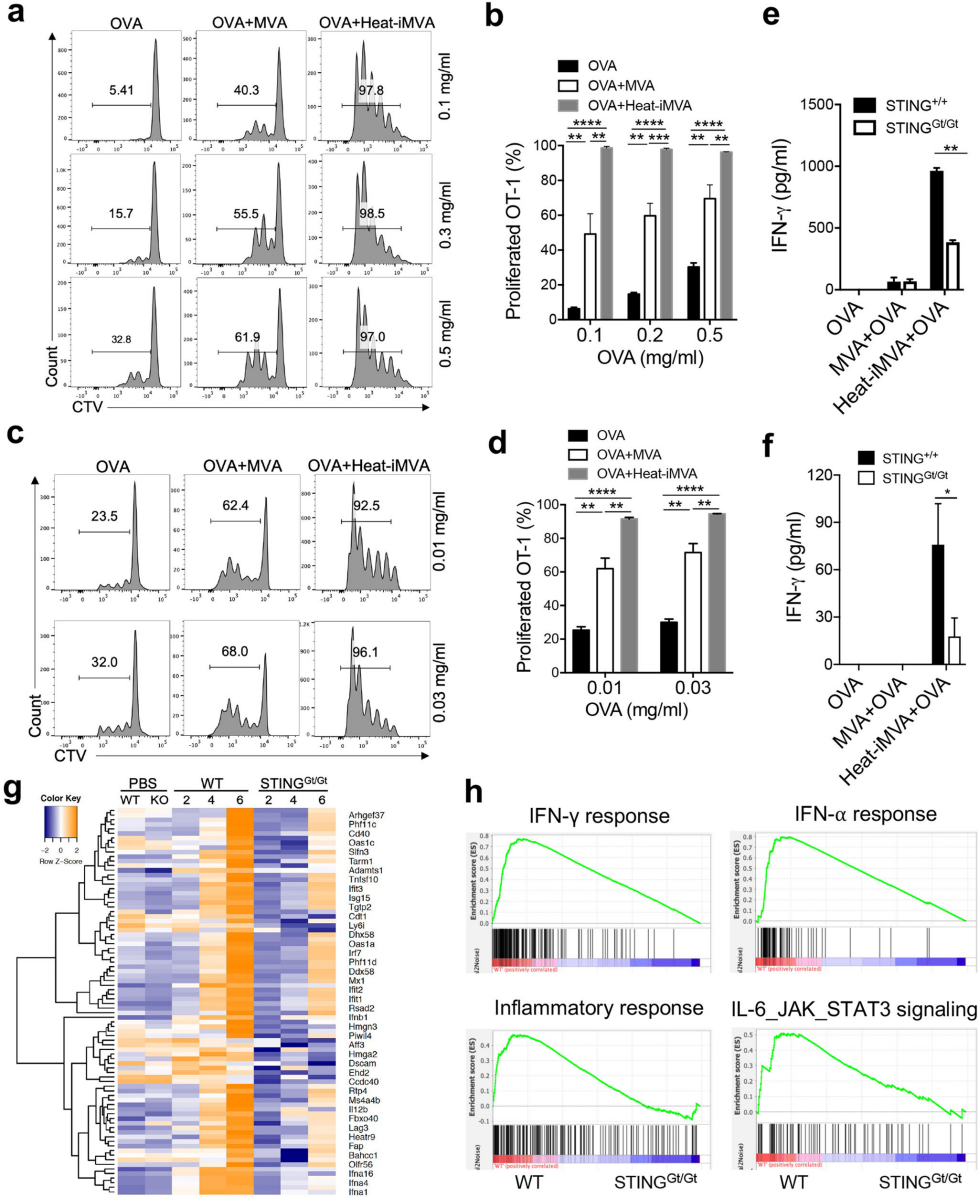
Heat-iMVA promotes OT-I cell activation and proliferation mediated by OVA cross-presentation by dendritic cells in vitro. **a**–**d** Proliferation of CTV-labeled OT-Ι T cells after incubation with GM-CSF-cultured BMDCs (**a**, **b**) or FLT3L-cultured dendritic cells (**c**, **d**) pulsed with OVA in the presence or absence of MVA or heat-iMVA. BMDCs were incubated with or without MVA or heat-iMVA, and then co-cultured with CTV-labeled OT-Ι cells for 3 days. **e** IFN-γ secretion from OT-Ι T cells after incubation with GM-CSF-cultured WT or STING^Gt/Gt^ BMDCs pulsed with OVA in the presence or absence of live MVA or heat-iMVA. **f** IFN-γ secretion from OT-Ι T cells after incubation with sorted CD103^+^ DCs from WT or STING^Gt/Gt^ Flt3L-cultured BMDCs pulsed with OVA in the presence or absence of live MVA or heat-iMVA. **g** A heat map of a one-way hierarchical clustering analysis of the differentially expressed genes between WT or STING^Gt/Gt^ BMDCs treated with Heat-iMVA over time (2, 4, and 6 h). **h** Gene set enrichment analyses (GSEA) showing differences of gene expression in several pathways including IFN-γ, IFN-α, inflammatory responses, and IL-6_JAK_STAT3 signaling in WT and STING^Gt/Gt^ BMDCs infected with heat-iMVA. Data are represented as mean ± SEM (*n* = 3-5; ^**^P < 0.01 and ^***^P < 0.001; unpaired multiple *t* test). Data are representative of three independent experiments.

Infection of epidermal DCs with live VACV inhibits DCs’ capacity to promote the proliferation of antigen-specific T-cells^42^. To test whether heat-iMVA or live MVA infection of BMDCs enhances antigen cross-presentation and the proliferation of antigen-specific OT-I T cells, we incubated GM-CSF-cultured BMDCs with OVA at various concentrations in the presence or absence of heat-iMVA or live MVA for 3 h. Cells were then washed to remove OVA or viruses and co-cultured with CellTrace violet (CTV)-labeled OT-Ι T cells for 3 days. OT-I cells recognize the OVA_257-264_ (SIINFEKL) peptide presented on MHC-I and proliferate under the influence of T cell receptor (TCR) stimulation and inflammatory cytokines. OT-I cell proliferation was quantified by measuring CTV intensities by FACS, which showed that infection of GM-CSF-cultured BMDCs with heat-iMVA enhanced the capacity of BMDCs to stimulate the proliferation of OT-I T-cells at all tested OVA concentrations (0.1, 0.3, and 0.5 mg/ml), as indicated by CTV dilution in the dividing cells (Fig. 4a, b). Live MVA infection of BMDCs also promoted antigen cross-presentation and proliferation of OT-1 cells, but it was less potent than heat-iMVA (Fig. 4a, b).

FMS-like tyrosine kinase 3 ligand (Flt3L) is a growth factor critical for the differentiation of Batf3-dependent CD103^+^/CD8α^+^ DCs and plasmacytoid DCs (pDCs). Flt3L-cultured BMDCs were pulsed with OVA in the presence or absence of heat-iMVA or live MVA, and then co-cultured with CFSE-labeled OT-Ι cells for 3 days. Heat-iMVA more potently stimulated Flt3L-BMDCs’ abilities to cross-present OVA and promote the proliferation of OT-I cells than MVA, even at an OVA concentration of 0.01 mg/ml (Fig. 4c, d).

To test whether the cGAS/STING-mediated cytosolic DNA-sensing pathway plays a role in antigen cross-presentation by BMDCs, we incubated WT or STING^Gt/Gt^ GM-CSF-cultured BMDCs with OVA in the presence or absence of either live MVA or heat-iMVA for 3 h. We then washed away OVA and viruses and co-cultured the OVA-pulsed BMDCs with OT-1 cells for 3 days. IFN-γ levels in the supernatants were determined by ELISA. We observed that GM-CSF-cultured BMDCs infected with heat-iMVA were more potent in cross-presenting OVA antigen and stimulating IFN-γ secretion from proliferated and activated OT-I cells than BMDCs infected with live MVA (Fig. 4e). However, IFN-γ levels were much lower in STING-deficient DCs than in WT DCs pretreated with heat-IMVA plus OVA and co-cultured with OT-I cells (Fig. 4e). To test whether the cGAS/STING pathway is important for heat-iMVA-induced antigen cross-presentation in CD103^+^ DCs, we sorted CD103^+^ DCs from Flt3L-cultured BMDCs from WT or STING^Gt/Gt^ mice and infected them with heat-iMVA. The cells were then pulsed with OVA for 3 h before they were washed and co-incubated with OT-1 cells for 3 days. IFN-γ levels were much lower in STING-deficient CD103^+^ DCs than WT CD103^+^ DCs (Fig. 4f). These results indicate that the cGAS/STING pathway plays a vital role in heat-iMVA-induced, CD103^+^ DCs-mediated antigen cross-presentation and antigen-specific T cell proliferation and activation.

### Heat-iMVA infection of BMDCs does not increase phagocytosis of soluble antigen

Infection of BMDCs with heat-iMVA induces DC maturation that is dependent on the STING-mediated cytosolic DNA-sensing pathway^25^. To assess whether BMDCs’ capacity for uptake of fluorescent Alexa Fluor 647-labeled model antigen OVA (OVA-647) is affected by heat-iMVA treatment, BMDCs were infected with heat-iMVA (with an equivalent MOI of 1) for 1 h and then incubated with OVA-647 for 1 h. The fluorescence intensities of phagocytosed OVA-647 in BMDC were then measured by flow cytometry, showing that pre-incubation of BMDCs with heat-iMVA for 1 h did not affect their capacity to phagocytose OVA-647 (Supplemental Fig. 1a, b). These results indicate that upon heat-iMVA infection didn’t increase phagocytosis of soluble antigen in BMDCs.

### Heat-iMVA infection of BMDCs induces STING-dependent IFN and inflammatory cytokine responses

To probe host transcriptomic changes induced by live MVA or heat-iMVA infection of BMDCs and to assess the contribution of the STING-mediated cytosolic DNA-sensing pathway in this process, we performed RNA-seq analyses of BMDCs from WT and STING^Gt/Gt^ mice infected with either live MVA or heat-iMVA for 2, 4, and 6 h. Our results showed several patterns of gene expression induced by MVA and heat-iMVA: (i) Infection with live MVA, but not heat-iMVA, induced a subset of host genes in a STING-independent manner, thus indicating gene induction by a live virus infection (Supplemental Fig. 2a, marked as a1-2); (ii) Heat-iMVA induced higher levels of a large subset of IFN-regulated genes than live MVA, which were mainly dependent on STING (Supplemental Fig. 2a, marked as b1-3); and (iii) Heat-iMVA infection induced higher levels of a relatively small subset of genes than MVA, which were independent of STING (Supplemental Fig. 2a, marked as c). Selected examples of genes in each category are shown (Fig. 4g and Supplemental Fig. 2b). For example, heat-iMVA infection triggered higher levels of IFN-inducible genes than MVA, including *Ifih1 (MDA5), Ddx58 (RIG-1), Oasl2, Oas3, TLR3, Nod1, Ifna4, Ifnb1, Ccl5, Cxcl9, Cxcl10*, and members of the guanylate binding protein (Gbp) family, as largely dependent on STING (Supplemental Fig. 2b, marked as b). These results indicate that the activation of the cytosolic DNA-sensing pathway triggers the up-regulation of genes involved in the cytosolic RNA-sensing pathway in addition to other antiviral genes, thereby strengthening host defense against viral invasion.

MVA infection of BMDCs resulted in the temporal expression of viral RNAs, as shown by the unbiased hierarchical cluster analysis (Supplemental Fig. 2c). Our results demonstrated that a large set of genes are expressed early during infection, consistent with published results of RNAseq of VACV-infected HeLa cells^43^. By contrast, heat-iMVA infection of cDCs did not result in significant levels of viral transcripts detected by the RNA-seq method (Supplemental Fig. 2c). Gene Set Enrichment Analyses (GSEA) confirmed that heat-iMVA-induced IFN-α, IFN-γ, inflammatory responses, and IL-6/JAK/STAT3 signaling in WT BMDCs but not in STING-deficient DCs (Fig. 4h). Together, RNA-seq analyses showed that live MVA and heat-iMVA infection trigger distinctive host and viral transcriptomic profiles in DCs, and that heat-iMVA is more immune-stimulatory likely due to the lack of expression of viral inhibitory genes. In addition, heat-iMVA-induction of type I IFN and IFN stimulated genes (ISGs) is largely dependent on STING.

### Heat-iMVA promotes migratory DC trafficking and maturation of resident DCs in the draining lymph nodes

**Fig. 5:**
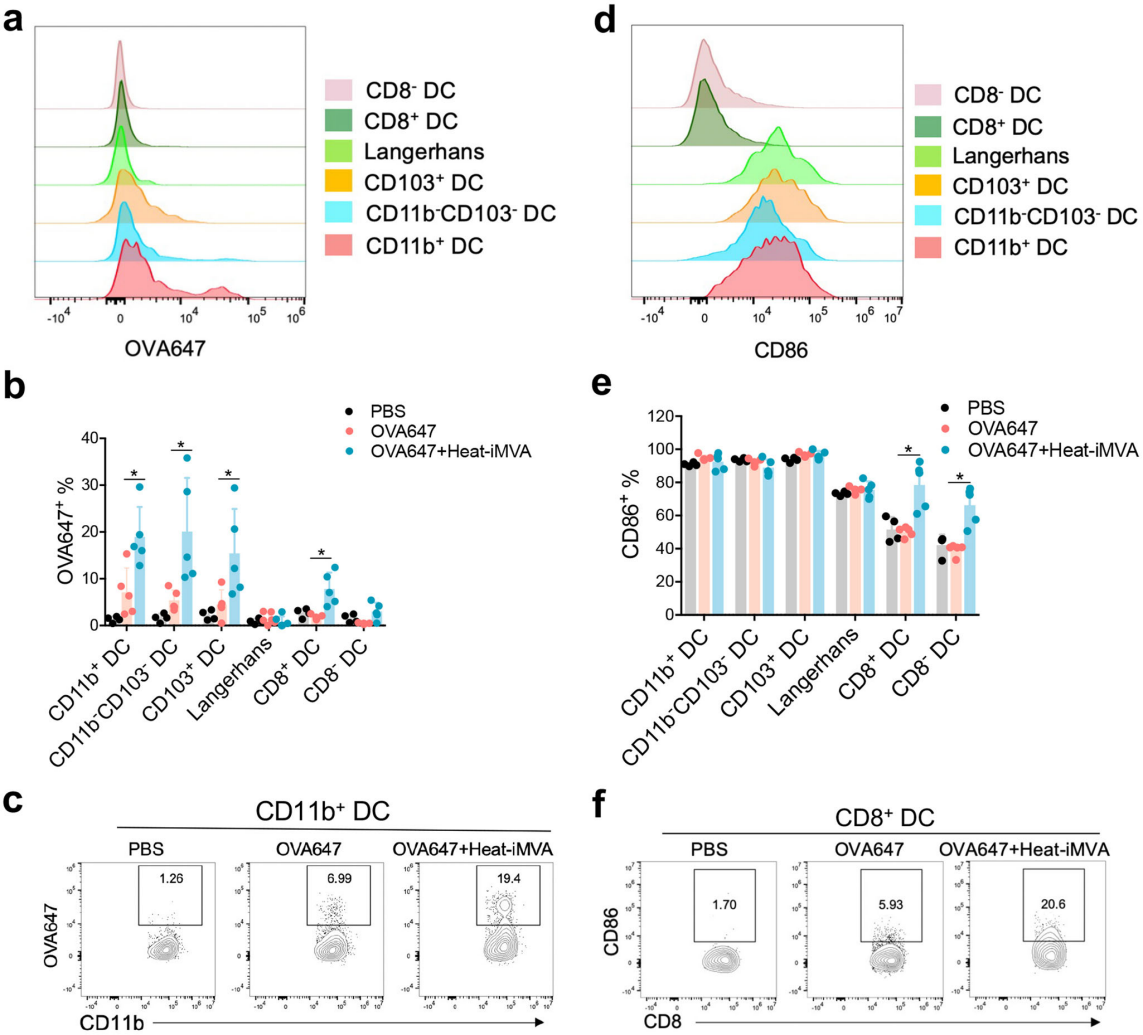
Co-administration of heat-iMVA promotes trafficking of antigen-carrying migratory DC into skin draining LN and activation of resident dendritic cells. C57/B6J mice were intradermally vaccinated with OVA647 (5 μg) in the presence or absence of heat-iMVA (10^7^ pfu). **a**, **b** After 24 h, OVA647 intensities in different dendritic cells populations from dLNs were measured. **c** Representative dot plots of OVA647 expression in CD11b^+^ dendritic cells. **d**, **e** After 24 h, CD86 expressions in different dendritic cells populations from dLNs were measured. **f** Representative dot plots of CD86 expression in CD8^+^ dendritic cells. Data are represented as mean ± SEM (*n* = 3-5; ^*^P < 0.05, and ^**^P < 0.01; Two-tailed Mann-Whitney *U* test). Data are representative of three independent experiments.

The DC lineage is heterogeneous and composed of migratory and resident DCs^44^. Migratory DCs capture antigens in the peripheral tissue and then mature, followed by migration to the draining lymph nodes, where they present antigens to naïve T cells. They can also transfer some antigens to resident DCs^45,46^. To elucidate the contributions of skin DC subsets in heat-iMVA-induced immune response, we analyzed various DC populations in skin draining lymph nodes after vaccination using a similar gating strategy as reported^47^. First, we were able to confirm six distinct DC populations in the skin draining lymph nodes (dLNs): (i) MHC-ΙΙ^+^CD11c^+^ migratory DCs and MHC-ΙΙ^Int^CD11c^+^ resident DCs; (ii) migratory DCs further separated into CD11b^+^ DCs, Langerin^−^ CD11b^−^ DCs, and Langerin^+^ DCs, which are CD103^+^ DCs and Langerhans cells; and (iii) resident DCs composed of CD8α^+^ lymphoid-resident DCs and CD8α^−^ lymphoid-resident DCs (Supplemental Fig. 3). Second, we tested which DCs subsets efficiently phagocytosing OVA antigen labeled with an Alexa Fluor 647 dye (OVA-647) and have the capacity to migrate to the skin dLNs. We intradermally injected OVA-647 into the right flanks of mice and harvested the skin dLNs at 24 h post injection. Consistent with a previous report showing that migratory DCs are responsible for transferring antigens to skin draining LN^48^, we observed that OVA-647 was mostly found in the three types of migratory DCs, including CD11b^+^, CD103^+^, and CD11b^−^CD103^−^ DCs, but rarely detected in resident DCs (Fig. 5a). To compare whether co-administration of OVA-647 with or without heat-iMVA affects OVA-647^+^ uptake by the migratory DCs and potential transfer to resident DCs in the skin dLNs, we intradermally injected OVA-647 with or without heat-iMVA and analyzed OVA-647^+^ DCs in the skin dLNs. Co-administration of OVA-647 with heat-iMVA increased the percentages of OVA-647^+^ CD11b^+^, CD103^+^, CD11b^−^CD103^−^, and CD8α^+^ DCs, compared with injection of OVA-647 alone (*P* < 0.05; *n* = 5; OVA + Heat-iMVA vs. OVA, Fig. 5b, c). These results suggest that co-administration of OVA-647 with heat-iMVA enhances the capacity of migratory DCs to transport phagocytosed antigen to the skin dLNs and facilitates the antigen transfer from migratory DCs to CD8α^+^ DCs, a lymphoid-resident DC population critical for antigen cross-presentation.

We also evaluated DC maturation status in the skin dLNs using CD86 as a maturation marker. Migratory DCs expressed higher levels of CD86 than resident DCs (Fig. 5d, e). Moreover, intradermal vaccination with OVA plus heat-iMVA induced higher levels of CD86 on resident DCs (CD8^+^ DC or CD8^−^ DC) than with OVA alone (Fig. 5e, f). By contrast, heat-iMVA co-administration did not change the maturation status of migratory DCs entering skin draining LNs (Fig. 5e). Our results indicate that intradermal co-administration of heat-iMVA with OVA antigen promotes antigen-carrying migratory DCs trafficking into the skin draining LN and transferring of antigens from migratory DCs to CD8α^+^ DCs and induce resident DC maturation. These effects contribute to heat-iMVA’s ability to induce antigen-specific adaptive immune responses.

### Co-administration of tumor neoantigen peptides with heat-iMVA improves antitumor effects in a murine therapeutic vaccination model

**Fig. 6:**
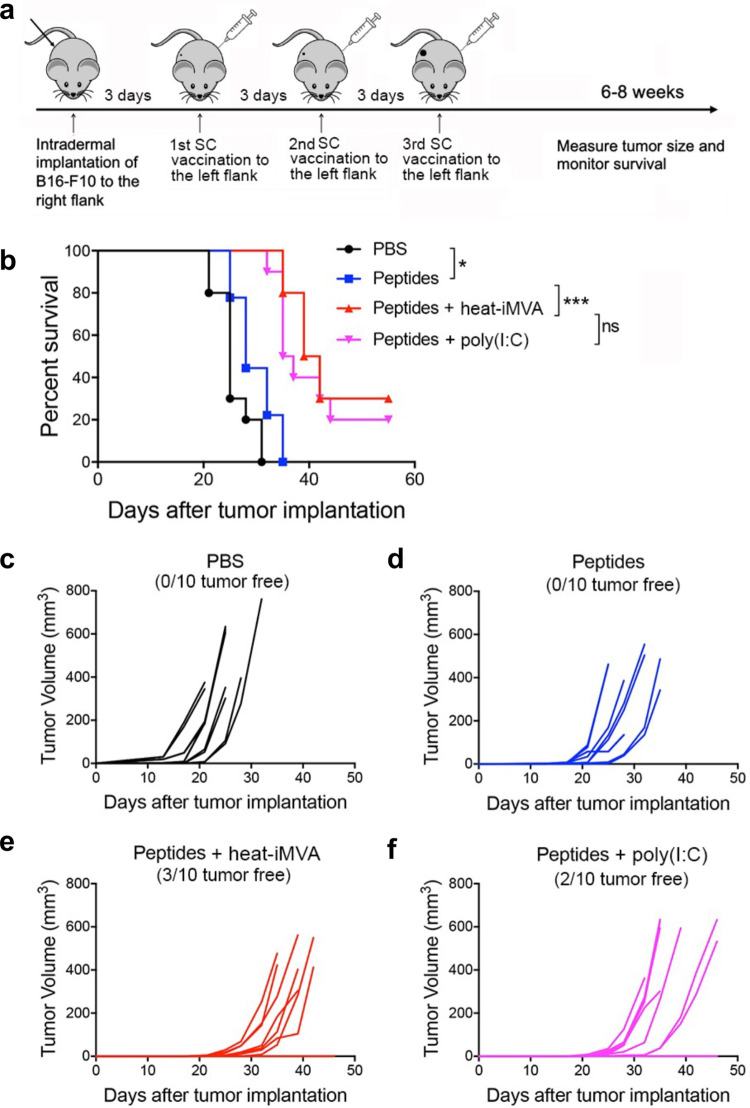
Combination of B16-F10 neoantigen peptides with heat-iMVA vaccination significantly increases the overall response and cure rates in a unilateral B16-F10 implantation model. **a** Tumor implantation and neoantigen peptide vaccination scheme in a unilateral B16-F10 tumor implantation model. 5 × 10^4^ B16-F10 were intradermally implanted into the right flanks of C57BL/6J mice. On day 3, 6, and 9, mice were vaccinated subcutaneously on the left flanks with B16-F10 neoantigen peptide mix (M27, M30, and M48) with or without the indicated adjuvants. **b** Kaplan–Meier survival curve of tumor-bearing mice treated with PBS, peptides (M27, M30, and M48, 100 μg/each), peptides plus heat-iMVA (an equivalent of 10^7^ pfu), or peptides plus poly(I:C) (50 μg) (*n* = 10, ^*^P < 0.05 and ^***^P < 0.001; Mantel–Cox test). **c**–**f** Tumor volumes over days after implantation in mice vaccinated with PBS (**c**), peptides (**d**), peptides + heat-iMVA (**e**), peptides + poly(I:C) (**f**). Data are representative of two independent experiments.

Tumor neoantigens are promising treatment targets, and neoantigen-based cancer vaccines have been intensively investigated in preclinical studies and clinical trials^49^. However, vaccination with neoantigen peptides alone induces only weak immune responses, thus limiting this approach. To overcome this weak vaccination efficiency, mRNA-based neoantigen vaccination or neoantigens peptides adjuvanted with poly(I:C) have shown some success^5,6^. Here, we tested whether therapeutic vaccination with neoantigen peptides plus heat-iMVA would delay tumor growth in a murine B16-F10 melanoma model. Three days after B16-F10 cells were implanted, we subcutaneously co-administered melanoma neoantigen peptides (M27, M30, and M48) plus heat-iMVA three times, three days apart, and monitored tumor growth and mouse survival (Fig. 6a). Neoantigen peptides alone only minimally delayed tumor growth, with an extended median survival from 25 days in the PBS group to 28 days in the peptides alone group (*P* < 0.05; *n* = 10; peptides vs. PBS, Fig. 6b–d). However, co-administration of neoantigen peptides with heat-iMVA cured B16-F10 melanoma in 30% of treated mice and prolonged the median survival from 28 days in the peptides alone group to 41 days in the peptides + heat-iMVA group (*P* < 0.0001; *n* = 10; peptides vs. peptides + heat-iMVA, Fig. 6b, d, e). Likewise, co-administration of poly(I:C) (50 μg) with neoantigen peptides also improved therapeutic vaccine efficacy with potency similar to heat-iMVA (Fig. 6b, d, f). We did, however, observe side effects, including weight loss in the poly(I:C) group (but not with heat-iMVA), likely due to systemic inflammatory responses to poly(I:C). These results indicate that heat-iMVA could be a safe and potent vaccine adjuvant that eradicates or delays tumor growth in a murine therapeutic vaccination model.

### Co-administration of heat-iMVA with SARS-CoV2 spike protein promotes robust neutralizing antibody production

**Fig. 7:**
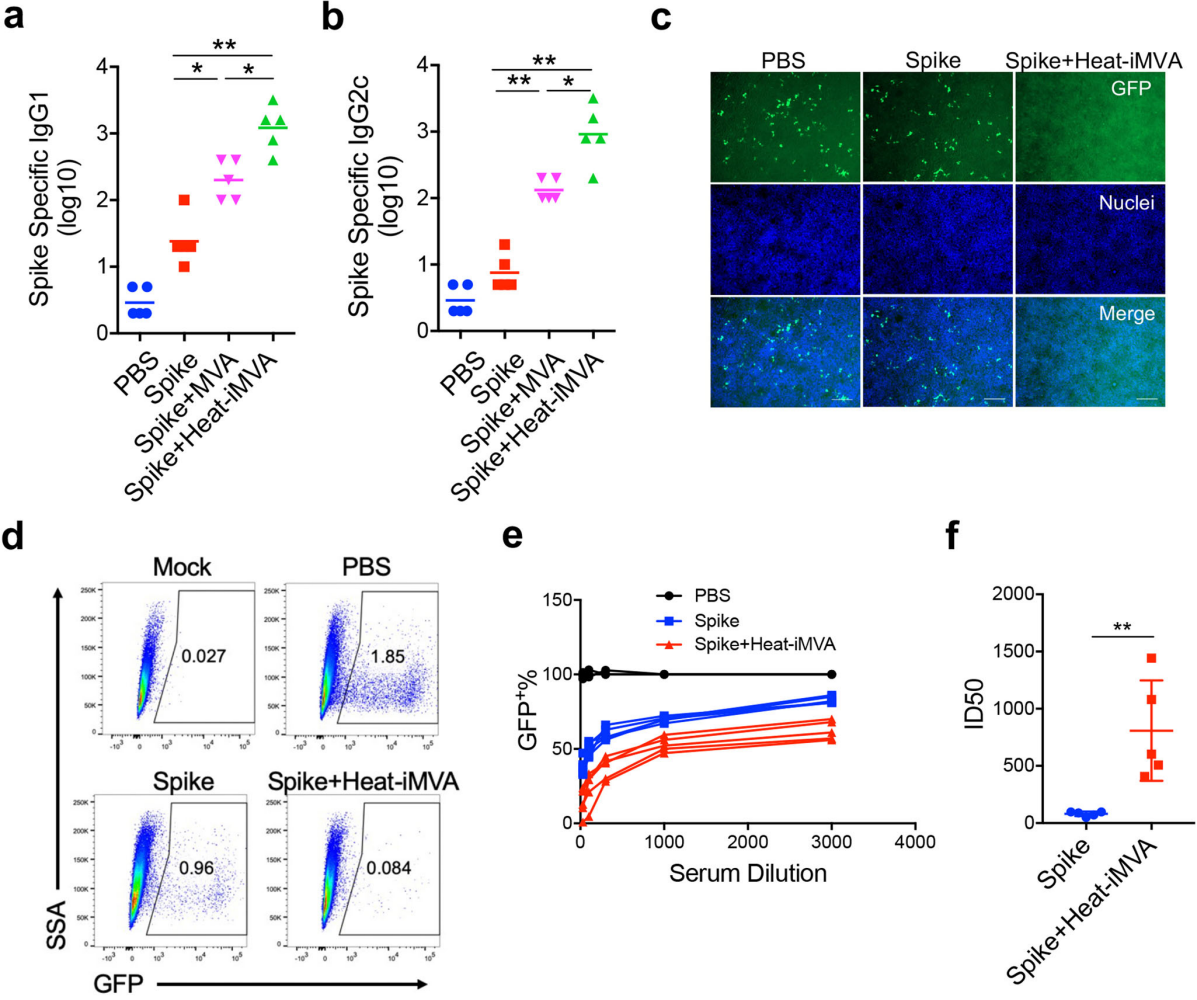
Heat-iMVA promotes stronger Th1 responses and IgG1, IgG2c production after intramuscular (IM) vaccination with SARS-CoV2 full length spike protein. WT C57BL/6J mice were vaccinated at day 0 and day 21 with SARS-CoV2 Spike (1 μg), spike (1 µg) + MVA (10^7^ pfu) or Spike (1 μg) plus Heat-iMVA (10^7^ pfu) intramuscularly. **a**, **b** Spike-specific immunoglobulin G1 (IgG1) or Spike-specific immunoglobulin G2c (IgG2c) titers in the serum from PBS, Spike, or Spike plus Heat-iMVA-vaccinated mice one week after second vaccination were determined by ELISA. **c**–**f** HEK293T-ACE2 cells were infected with SARS-CoV-2 pseudovirus at the presence of mouse serum (1:100 dilution). After 48 h, spike protein mediated virus entry was detected by GFP expression. **c** GFP was observed by fluorescence microscope. **d** GFP was measured by FACS analysis. **e** The neutralizing antibodies in serum at different dilution was detected by GFP expression based on FACS analysis. **f** 50% inhibitory dose (ID50) was determined. Data are represented as mean ± SD (*n* = 3–5). **p < 0.01 and ****p < 0.0001 (Two-tailed Mann–Whitney *U* test). Data are representative of two (**a**, **b**) or three (**c**–**f**) independent experiments.

Effective vaccines against SARS-CoV-2 are urgently needed to control the global pandemic of COVID-19 in developing nations^50^. SARS-CoV-2 spike protein is a particularly attractive target for vaccine design^51,52^ and mRNA vaccines encoding an optimized spike protein (BNT162b2 and mRNA-1273) are now approved for emergency use. However, spike protein alone is only weakly immunogenic, limiting its application as a subunit vaccine. Here, we tested whether co-administration of recombinant spike protein with heat-iMVA generates anti-spike neutralizing antibodies. C57BL/6J mice were vaccinated intramuscularly with either recombinant spike protein alone or spike + heat-iMVA twice, three weeks apart, with serum collected at one week post-second vaccination. Our results showed that vaccination with spike protein alone slightly induced anti-spike IgG1 and IgG2c antibodies (Fig. 7a, b), while co-administration of spike protein + heat-iMVA increased IgG1 levels by 50-fold and IgG2c levels by 100-fold compared with vaccination with spike protein alone (Fig. 7a, b). Although MVA also boosted spike-specific IgG responses, the IgG1 and IgG2c levels were lower than those induced by heat-iMVA (Fig. 7a, b).

To investigate whether vaccination-induced antibodies could block SARS-CoV-2 infection, we performed a neutralization assay using a SARS-CoV-2 pseudovirus. Without any pretreatment, the SARS-CoV-2 pseudovirus carrying the gene encoding GFP efficiently infected human ACE2-expressing HEK293T cells, as shown by the GFP^+^ cells (Fig. 7c). Pretreatment with serum from the spike + heat-iMVA group at 1:100 dilution efficiently blocked SARS-CoV-2 pseudovirus infection, whereas pretreatment with serum from the spike alone group only weakly reduced pseudovirus infection (Fig. 7c). Flow cytometry analysis of GFP^+^ cells confirmed our observation (Fig. 7d). Serum neutralizing antibody titers in the two vaccination groups and a PBS-mock vaccination group was determined (Fig. 7d, e). ID50 (50% inhibitory dose) was defined as the reciprocal of the serum dilution that caused a 50% reduction of GFP^+^ cells compared with mock-treated samples. The serum neutralizing antibody titers (ID50) from spike + heat-iMVA group were 10-fold higher than those from the spike alone group (Fig. 7f). Overall, our results indicate that heat-iMVA boosts the production of neutralizing antibodies when combined with the recombinant spike protein from SARS-CoV-2.

## Discussion

In this study, we explored the use of heat-iMVA as a vaccine adjuvant for protein- or peptide-based subunit vaccines against cancers and infectious agents. MVA is an approved vaccine against smallpox, and a potential viral vector with an excellent safety profile. A typical feature of MVA is that, comparing to CVA, its ancestor, and other VACV strains, it has lost immunosuppressive genes that allow stimulation of innate immunity. However, it still expresses some immune-suppressive genes, such as E3L. We have previously shown that heat-inactivated vaccinia enters DCs via its entry-fushion complex composed of multiple vaccinia proteins including A28^30^. Similar to heat-inactivated vaccinia virus, Heat-iMVA most likely preserves the ability to enter DCs, but fails to express viral genes, thus inducing much higher levels of type I IFN and ISGs than live MVA via the cGAS/STING-mediated cytosolic DNA-sensing pathway. Here, we demonstrated that co-administration of heat-iMVA with soluble proteins or peptides generates Th1-biased cellular and humoral immune responses superior to known adjuvants, including CFA and AddaVax. In a murine therapeutic vaccination model, co-delivery of heat-iMVA with three B16-F10 neoantigen peptides delayed tumor growth and cured 30% of tumor-bearing mice, with similar efficacy as poly(I:C), but with less toxicity. Furthermore, vaccination with heat-iMVA plus SARS-CoV-2 spike protein potently induced neutralizing antibodies. Taken together, our results support the use of heat-iMVA as a vaccine adjuvant.

DCs are essential for priming naïve T cells to generate adaptive immune responses, and therefore are the primary targets of vaccine adjuvants^14,53–56^. RNA-seq analyses of host transcriptomes of DCs infected with either live MVA or heat-iMVA revealed heat-iMVA as a more potent STING agonist than live MVA, inducing large subsets of genes involved in type I and type II IFN and inflammatory responses. We surmise that viral DNA from heat-inactivated MVA virion particles are detected by the cytosolic DNA sensor cGAS leading to the production of second messenger cyclic GMP-AMP (cGAMP), which then activates the STING pathway^31^.

We previously showed that heat-iMVA infection of BMDCs induced DC maturation in a STING-dependent manner^31^. Consistent with this, we now find that heat-iMVA infection of BMDCs promotes antigen cross-presentation, which requires STING. Barnowski et al.^57^ showed that the STING pathway contributes to the generation of VACV immunodominant B8-specific CD8^+^ T cell, but not to anti-OVA CD8^+^ T cell responses, after intraperitoneal vaccination with MVA expressing OVA. Together, these results show that the STING pathway is involved in MVA-induced antiviral adaptive immunity as well as the MVA-mediated adjuvant effect.

Using Batf3^−/−^ mice, we also demonstrated that heat-iMVA-boosted antigen-specific CD8^+^ T cell responses are dependent on cDC1s, also known as CD103^+^/CD8α^+^ DCs. However, heat-iMVA-boosted antigen-specific CD4^+^ T cell responses were not lost in Batf3^−/−^ mice, suggesting that cDC2s, also known as CD11b^+^ DCs, might be responsible for antigen presentation via MHC-II. Interestingly, heat-iMVA-boosted antigen-specific antibody responses were not affected in Batf3^−/−^ mice. This finding is consistent with a recent report that migratory CD11b^+^ DCs (cDC2s) are required for priming T follicular helper (Tfh) cells, a subset of CD4^+^ T cells, for antigen-specific antibody production^58^.

An increasing body of evidence indicates that STING agonists can function as potent vaccine adjuvants^12,13,41,59–61^. To probe the role of the STING-mediated cytosolic DNA-sensing pathway in heat-iMVA adjuvanticity, we used STING^Gt/Gt^ mice, which lack functional STING^62^. Our results demonstrate that STING contributes to the generation of antigen-specific IFN-γ^+^ CD8^+^ and CD4^+^ T cells and IgG2c antibody production potentiated by heat-iMVA. Given the essential roles of cDC1 and cDC2 in mediating CD8^+^ and CD4^+^ T cell priming, we surmise that STING signaling in cDC1 and cDC2 might be important for heat-iMVA-induced immunogenicity.

To further delineate the mechanisms of action of heat-iMVA as a vaccine adjuvant, we evaluated whether intradermal co-delivery of heat-iMVA and OVA-647 (OVA-conjugated with Alex Fluor 647) increases migratory DC trafficking to the dLNs compared with OVA-647 alone. Our results showed that heat-iMVA co-administration significantly increased OVA-647^+^ migratory DCs in the dLNs, which include CD11b^+^, CD103^+^, and CD11b^−^CD103^−^ DCs. Interestingly, heat-iMVA also increased OVA-647^+^ CD8α^+^ DCs in the dLNs. Although migratory DCs in the dLNs exhibited high CD86 expression with or without heat-iMVA as a vaccine adjuvant, heat-iMVA co-delivery resulted in higher expression of CD86 on resident DCs, including both CD8^+^ DCs and CD8^−^ DCs. These results suggest that heat-iMVA co-administration promotes peripheral DC maturation and migration into dLNs, as well as LN- resident DC maturation. We speculate that some heat-iMVA virions (together with OVA-647) might be transported, via the LN conduits, to the LN interior to resident DCs, as demonstrated for subcutaneously injected VACV virus or MVA^63^.

IM is the most commonly used vaccination route for approved vaccines and SC has been investigated for new vaccine platforms due to its skin-targeting effects. Therefore, they were used for most of the experiments in this study. We found that the cellar and humoral immune responses were similar between the two vaccination routes. In fact, several clinical trials comparing vaccine efficacy between IM and SC immunization also showed no significant difference between the two^64–66^. A recent study by Ols et al. using nanoparticle vaccine administered via IM or SC routes in rhesus macaques also did not observe significant differences in the magnitude and quality of adaptive immune responses, although antigen distributions differed^67^. Although skin scarification (SS) might be an efficient vaccination route to deliver MVA^68,69^, we excluded this route of vaccination because SS delivery of protein or peptide antigens may not be ideal. We also did not include intranasal vaccination for this study due to similar concerns, although intranasal vaccination with lyophilized MVA has been shown to generate long-lasting protective immunity^70^.

Because the skin dermis has enriched dendritic cell populations^71^, we performed intradermal (ID) vaccination approach to evaluate whether co-administration of heat-iMVA with OVA influence the dynamics of antigen presentation in the skin draining LNs. Our results demonstrate that ID delivery of heat-iMVA and OVA promotes trafficking of antigen-carrying migratory DC subsets to the skin draining LNs and antigen transfer between migratory DCs to the resident DCs in the skin draining LNs.

We envision that heat-iMVA can be used as a vaccine adjuvant for neoantigen-based cancer vaccines based on its safety and immunogenicity. Moreover, we demonstrate that heat-iMVA activation of STING signaling contributes to its adjuvanticity and heat-iMVA promotes antigen cross-presentation by Batf3-dependent CD103^+^/CD8α^+^ DCs to induce antigen-specific CD8^+^ T cell responses. Nörder et al.^72^ investigated whether MVA could be used as a vaccine adjuvant, and they found that IM co-administration of MVA and OVA enhances the generation of antigen-specific antibody and T cell responses. Here we provided evidence that heat-iMVA is a more potent vaccine adjuvant than live MVA. Future work would focus on identifying viral inhibitors of the cGAS/STING pathway encoded by the MVA genome and engineering recombinant MVA to improve its immunogenicity and adjuvanticity.

## Methods

### Mice

Female C57BL/6J mice between 6 and 8 weeks of age were purchased from the Jackson Laboratory and were used for vaccination experiments and for the preparation of bone marrow-derived dendritic cells. Batf3^−/−^ mice were generated in the laboratory of Kenneth Murphy (Washington University). STING^Gt/Gt^ mice were generated in the laboratory of Russell Vance (University of California, Berkeley). OT-1 mice were generated in the laboratory of Michael Bevan (University of Washington) and purchased from the Jackson laboratory. All mice were maintained in the animal facility at the Sloan Kettering Cancer Institute. All procedures were performed in strict accordance with the recommendations in the *Guide for the Care and Use of Laboratory Animals* of the National Institute of Health. The protocol was approved by the Committee on the Ethics of Animal Experiments of Sloan-Kettering Cancer Institute.

### Cell lines and primary Cells

BHK-21 was cultured in Eagle’s Minimal Essential Medium (Eagle’s MEM, Life Technologies, Cat# 11095-080) containing 10% FBS, 0.1 mM nonessential amino acids, and 1% penicillin-streptomycin. BHK-21 cells were regularly monitored for potential bacterial contamination under the microscope before virus preparation. For the generation of GM-CSF-BMDCs, bone marrow cells (5 million cells in each 15 cm cell culture dish) were cultured in RPMI 1640 medium supplemented with 10% fetal bovine serum (FBS) in the presence of murine GM-CSF (30 ng/ml, PeproTech) for 10–12 days. For the generation of fms-like tyrosine kinase-3 ligand (Flt3L)-cultured murine bone marrow-derived dendritic cells (Flt3L-BMDCs), bone marrow cells (5 × 10^6^ cells in each well of a six-well plate) were cultured in the presence of murine Flt3L (100 ng/ml, R & D Systems) for 7–9 days. Cells were fed every 2–3 days by replacing 50% of the old medium with fresh medium. HEK293T cell line expressing human ACE2 (hACE2) were generated by transduction with vesicular stomatitis virus (VSV) G protein-pseudotyped murine leukemia viruses (MLV) containing pQCXIP-hACE2-c9. Cells were selected and maintained in growth media containing 2 μg/ml puromycin for the selection of stably transduced cells. The murine melanoma cell line B16-F10 was originally obtained from I. Fidler (MD Anderson Cancer Center). Both cell lines were maintained in RPMI 1640 medium supplemented with 10% FBS, 1 × nonessential amino acids, 1 mM sodium pyruvate, 2 mM l-glutamine, 50 µM β-mercaptoethanol, and penicillin-streptomycin. Cells were regularly checked for potential contamination with mycoplasma with MycoAlert-Plus^TM^ kit (Lonza).

### Viruses

MVA and MVA-GFP viruses were kindly provided by Gerd Sutter (University of Munich) and propagated in BHK-21 (baby hamster kidney cell, ATCC CCL-10) cells. MVA and MVA-GFP stocks were prepared in BHK21 cells. Briefly, BHK21 cells in 15-cm plates were infected with MVA or MVA-GFP at MOI = 0.1. After 2 days or when cytopathic effect became obvious, cells were detached from the plates with a cell scraper, collected, and pelleted. After three cycles of freeze-thaw of resuspended cell pellets, they were homogenized using a cup sonicator with ice water. The virus materials were centrifuged, and the supernatants were further purified through a 36% sucrose cushion centrifugating at 17,000×*g* for 60 min at 4 °C. The purified virus pellet was resuspended in cold PBS and dispensed into 0.5 ml aliquots and stored at −80 °C. Heat-iMVA was generated by incubating purified MVA-GFP virus (in 0.5 ml aliquots) in a 55 °C water bath for 1 h with vertexing every 15 min. To determine MVA-GFP titer, serial dilutions of virus stock were prepared, and they were used to infect BHK-21 cells in six-well plates. After 48 h, the number of GFP foci was counted under the fluorescent microscope (Zeiss Axio Observer 7) and multiplied by the dilution factors to express titers as infectious units/ml. SARS-CoV-2 pseudoviruses were produced in HEK293T cells. Briefly, HEK293T cells were co-transfected with pQCXIG-SARS-CoV-2-Spike, pMD2.G (VSV-G) and a gag/pol expression plasmid. At 48 h post-transfection, virus supernatants were harvested and filtered through a 0.45-μm filter and stored at −80 °C.

### Reagents

EndoFit Ovalbumin, CFA, AddaVax, and poly(I:C) were purchased from InvivoGen. The SARS-CoV-2 spike protein was purchased from RayBiotech. Alexa FluorTM 647 conjugated OVA and CellTrace Violet were purchased from Thermo Fisher. B16-F10 tumor neoantigen peptides were synthesized by GenScript (Piscataway, NJ). The sequences are as follows: M27: REGVELCPGNKYEMRRHGTTHSLVIHD; M30: PSKPSFQEFVDWENVSPELNSTDQPFL; M48: SHCHWNDLAVIPAGVVHNWDFEPRKVS.

### OVA vaccination procedure

WT C57BL/6J mice were anesthetized and vaccinated initially on day 0 and boosted on day 14 with either OVA (10 µg) alone, OVA (10 µg) + MVA (10^7^ pfu) or OVA (10 µg) + heat-iMVA (an equivalent of 10^7^ pfu) in a volume 100 µl intramuscularly (IM) or subcutaneously (SC). Mice were euthanized on day 21. Spleens and blood were collected for analyzing OVA-specific CD8^+^, CD4^+^, and B cell responses. In some cases, OVA proteins were mixed with CFA or AddaVax. In some cases, STING^Gt/Gt^, Batf3^−/−^, and age-matched WT C57BL/6J mice were vaccinated with OVA + heat-iMVA.

### SARS-CoV-2 spike protein vaccination procedure

In all, 4–5 mice in each group were anesthetized and vaccinated with SARS-CoV-2 recombinant spike protein (1 µg) alone, spike (1 µg) + MVA (10^7^ pfu), or spike (1 μg) + heat-iMVA (an equivalent of 10^7^ pfu) in a volume 100 µl intramuscularly on day 0 and boosted on day 21. Mice were euthanized on day 28. Spike-specific immunoglobulin G1 (IgG1) or immunoglobulin G2c (IgG2c) titers in the serum from PBS, spike alone, spike + MVA or spike + heat-iMVA-vaccinated mice were determined by ELISA.

### Unilateral intradermal tumor implantation and therapeutic vaccination using neoantigen peptides with or without adjuvants

B16-F10 melanoma cells (5 × 10^4^) in a volume of 100 µl were implanted intradermally into the shaved skin on the right flank of WT C57BL/6 J mice. On day 3, 6, and 9, 4 groups of mice (10 mice in each group) were subcutaneously vaccinated at the left flanks with B16-F10 neoantigen peptide mix (M27, M30 and M48) (100 µg each), with or without heat-iMVA (an equivalent of 10^7^ pfu) or poly(I:C) (50 µg), or with PBS mock control, in a volume of 100 µl. Mice were monitored daily, and tumor sizes were measured twice a week. Tumor volumes were calculated according to the following formula: *l* (length) × *w* (width) × *h* (height)/2. Mice were euthanized for signs of distress or when the diameter of the tumor reached 10 mm.

### Flow cytometry analysis of antigen-specific T cells in the spleens

To analyze antigen-specific T cells in the spleens, spleens from vaccinated mice was collected and processed using Miltenyi GentleMACS™ Dissociator. Red blood cells were lysed using ACK lysing buffer (Lonza). For intracellular cytokine staining, splenic single-cell suspensions were stimulated with 10 μg/ml peptides (OVA_257__–__264_ or OVA_323__–__339_). After 1 h of stimulation, GolgiPlug (BD Biosciences; 1:1000 dilution) was added and incubated for 12 h. Cells were then treated with BD Cytofix/Cytoperm™ kit prior to staining with respective antibodies for flow cytometry analyses. The antibodies used for this assay are as follows: BioLegend: CD3e (145-2C11, cat# 100341), CD4 (GK1.5, cat# 100428), CD8 (53-5.8, cat# 140418), and IFN-γ (XMG1.2, cat# 505810). All antibodies were used at 1:200.

### Antibodies titer determination by ELISA

ELISA was used to determine anti-OVA or anti-SARS-CoV-2 spike IgG titers. Briefly, 96-well microtiter plates (Thermo Fisher) were coated with 2.0 µg/mL of OVA (Invivogen) or SARS-CoV-2 spike protein (RayBiotech) overnight at 4°C. Plates were washed with 0.05% Tween-20 in PBS (PBST) and blocked with 1% BSA/PBST. Mouse serum samples were two-fold serially diluted in PBST, added to the blocked plates, and incubated at 37 °C for 1 h. Following incubation, plates were washed with PBST and incubated with horseradish peroxidase (HRP)-conjugated goat anti-mouse IgG1 (Southern Biotech, cat#1070-05, 1:5000) or goat anti-mouse IgG2c (Southern Biotech, cat# PA1-29288, 1:4000) for 1 h. Plates were washed with PBST and TMB substrate (BD Bioscience) was added. Reactions were stopped with 50 µl 2 N H_2_SO_4_. Plates were read at OD 450 nm with a SpectraMax Plus plate reader (Molecular Devices). The antibody titer is defined as the dilution in which absorbance is more than 2.1 times of the blank wells.

### Flow cytometry analysis of migratory and skin LN-resident DCs after *fluorescent-labeled* OVA-647 vaccination with or without heat-iMVA

C57BL/6J mice were vaccinated intradermally at the right flank with either Alexa Fluor 647-labeled OVA (OVA-647, 10 µg) alone or OVA-647 (10 µg) + heat-iMVA (an equivalent of 10^7^ pfu) in a volume 100 µl PBS. Skin draining lymph nodes (dLNs) at the right inguinal area were harvested at 24 h post injection, digested with Collagenase D (400 U/ml, Roche Diagnostics) and DNase I (50 μg/ml, Roche Diagnostics), and analyzed by flow cytometry for OVA-647 intensities and CD86 expression of the migratory DC and resident DC populations in the skin dLNs. The antigens and clone designations for the antibodies are as follows: BioLegend: CD11c (N418, cat# 117320), CD11b (M1/70, cat# 101226), MHC-II (M5/114.15.2, cat# 107645), CD3e (145-2C11, cat# 100341), CD8a (53-6.7, cat# 140418); BD Biosciences: CD19 (1D3, cat# 562701), CD49b (DX5, cat# 563063), Thermo Fisher: CD16/CD32 (93, cat# 13-0161-82), CD103 (2E7, cat# 11-1031-82), CD207 (eBioL31, cat# 12-2075-82), and TER-119 (TER-119, cat# 48-5921-82). All antibodies were used at 1:200. Cells were analyzed on the BD LSR Fortessa or LSR II flow cytometer and data were analyzed with FlowJo software (version 10.5.3).

### RNA-seq analyses of GM-CSF-cultured BMDCs infected with live MVA vs. Heat-iMVA

GM-CSF-cultured BMDCs (1 × 10^6^) from WT or STING^Gt/Gt^ mice were infected with live MVA or heat-iMVA at a multiplicity of infection (MOI) of 10. Cells were collected at 2, 4, and 6 h post-infection. Total RNA was extracted using TRIzol (Thermo Fisher). Agilent 2100 Bioanalyzer at the Rockefeller University Genomics Resource Center was used to assess total RNA integrity and quantity. Samples with the RNA integrity number (RIN) > 9.5 were used.

Oligo(dT)-selected RNA was converted into cDNA for RNA sequencing using the Illumina TruSeq RNA Sample Preparation Kit v2 according to the instructions of the manufacturer and sequenced on an Illumina HiSeq 2500 platform using 100 nt single-end sequencing at The Rockefeller University Genomics Resource Center. Reads were aligned against the mouse genome (Gencode, GRCm38) plus MVA genome (GenBank: AY603355.1) using TopHat v2.0.14 (http://tophat.cbcb.umd.edu/). The steps described followed the protocol by Trapnell and colleagues (Trapnellet al.2012). Cufflinks v2.1.1 (http://cole-trapnell-lab.github.io/cufflinks/) was used for estimation of transcript abundance and differential expression analysis. Unsupervised hierarchical clustering was performed using Euclidean distance and complete linkage for columns (samples) and rows (mRNAs). For the sake of clarity, the row dendrograms were removed from the figures. The R packages pheatmap was used for data representation.

Gene set enrichment analysis (GSEA) was conducted using the package fgsea with 1000 permutations, with reactome and MSigDB C7 signature sets.

### Antigen cross-presentation assay

GM-CSF-cultured BMDCs or Flt3L-cultured BMDCs were infected with MVA or heat-iMVA at a MOI of 1 and then OVA was added at indicated concentrations and incubated for 3 h. Cells were washed away from virus and OVA, and co-cultured with CellTrace Violet (CTV)-labeled OT-1 for 3 days (BMDC:OT-1 T-cells =1:5). Flow cytometry was applied to measure CFSE intensities of OT-Ι cells.

WT or STING-deficient GM-CSF-cultured BMDCs were incubated with OVA in the presence or absence of either live MVA or heat-iMVA for 3 h. Cells were washed away from OVA and virus and co-cultured with OT-1 cells (BMDC to OT-1T-cells ratio of 1:3) for 3 days. IFN-γ levels in the supernatants were determined by ELISA (R&D).

OT-1 cells were purified from OT-1 transgenic mice using negative selection with CD8a^+^ T Cell Isolation Kit according to the manufacturer’s instructions (Miltenyi Biotec). Briefly, spleens and lymph nodes were harvested from OT-1 mice and mashed on 70-µM cell strainer. After red cell lysis, cells were resuspended in MACS buffer (PBS with 0.5% bovine serum albumin and 2 mM EDTA) and filtered through a 70-µM cell strainer. Biotin-antibody cocktail (including antibodies against CD4, CD11b, CD11c, CD19, CD45R (B220), CD49b (DX5), CD105, MHC Class II, Ter-119, and TCRγ/δ) was added to the cell mixture, and cells bound to the antibodies were removed using anti-biotin microbeads. The purified cells were resuspended in RPMI medium with 10% FCS.

### SARS-CoV-2 pseudovirus neutralization assay

Serially diluted serum was pre-incubated with SARS-CoV-2 pseudovirus at room temperature (RT) for 30 mins, and the mixtures were added to 293T-hACE2. Media was refreshed 2 h later. After 48 h, cells were fixed in 4% paraformaldehyde in PBS for 15 min at RT. Following three washes with PBS, the cell nuclei were stained with Hoechst 33258 (Sigma) in PBS for 10 min at RT. Images were captured using Zeiss Axio Observer 7 (Carl Zeiss) and analyzed with ZEN Imaging software (Carl Zeiss) and Image J (Fiji). GFP expression in pseudovirus-infected cells were determined using the BD LSR Fortessa flow cytometer and data were analyzed using FlowJo software (version 10.5.3).

### Statistics

Two-tailed Mann-Whitney *U* test was used for comparisons of two independent groups in the studies. Survival data were analyzed by log-rank (Mantel-Cox) test. The *P* values deemed significant are indicated in the figures as follows: *P < 0.05; **P < 0.01; ***P < 0.001; ****P < 0.0001. Statistical analyses were performed using the GraphPad Prism 7 Software. The numbers of animals included in the study are discussed in each figure legend.

### Reporting summary

Further information on research design is available in the Nature Research Reporting Summary linked to this article.

## Supplementary information

Heat inactivated modified vaccinia virus Ankara boosts Th1 cellular and humoral immunity as vaccine adjuvant

REPORTING SUMMARY

## Data Availability

RNA-sequencing data have been deposited at NCBI Short-Read Archive (SRA) and are publicly available as of the date of publication under the BioProject number PRJNA743347. All datasets generated and/or analyzed in this report are available from the corresponding author on reasonable request.
