# Supplementary material for: Heat-inactivated modified vaccinia virus Ankara boosts Th1 cellular and humoral immunity as a vaccine adjuvant
Source: NPJ Vaccines. 2022 Oct 19;7:120. doi: 10.1038/s41541-022-00542-5 (PMC9580433; doi:10.1038/s41541-022-00542-5)
Supplement: Supplementary file 1 — Heat inactivated modified vaccinia virus Ankara boosts Th1 cellular and humoral immunity as vaccine adjuvant [file 41541_2022_542_MOESM1_ESM.pdf]

Heat inactivated modified vaccinia virus Ankara boosts Th1 cellular and humoral immunity as vaccine adjuvant

Ning Yang[1], Aitor Garcia[2], Cindy Meyer[2], Thomas Tuschl[2], Taha Merghoub[3,4,5], Jedd D. Wolchok[3,4,5], and Liang Deng[1,3,4,5*]

[1]Dermatology Service, Department of Medicine, Memorial Sloan Kettering Cancer Center, New York, NY, 10065, USA

[2]Laboratory of RNA Molecular Biology, The Rockefeller University, New York, NY, 10065, USA

[3]Immuno-oncology service, Human Oncology and Pathogenesis Program; Memorial Sloan Kettering Cancer Center, New York, NY 10065, USA

[4]Parker Institute for Cancer Immunotherapy, Memorial Sloan Kettering Cancer Center, New York, NY, USA

[5]Weill Cornell Medical College, New York, NY, USA

*corresponding authors. Mailing address for Liang Deng: Dermatology Service, Department of Medicine, Memorial Sloan Kettering Cancer Center, 1275 York Ave., New York, NY 10065. Email: dengl@mskcc.org.

Short title: heat-inactivated MVA as a vaccine adjuvant

This file contains:

- Supplementary Figure 1
- Supplementary Figure 2
- Supplementary Figure 3

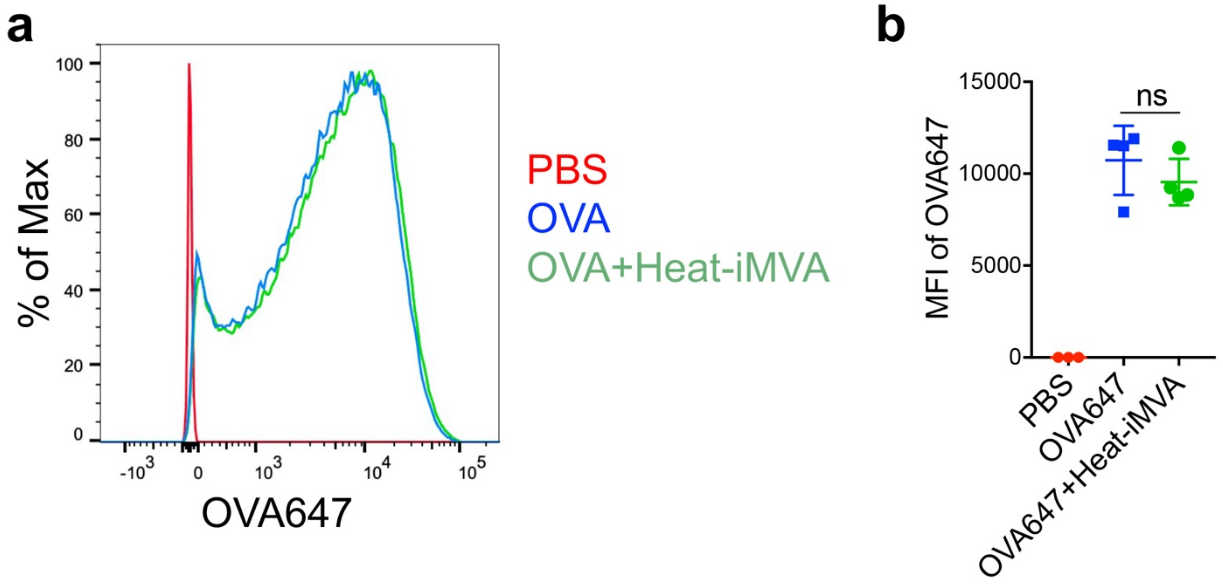

Supplementary Figure 1. Heat-iMVA infection of BMDCs does not enhance antigen uptake in vitro. (**a-b**) BMDC were infected with Heat-iMVA (MOI of 1) for 1 h and then incubated with OVA-647 (0.5 mg/ml) for 1 h. The fluorescence intensities of phagocytosed OVA-647 in BMDC were measured by flow cytometry.

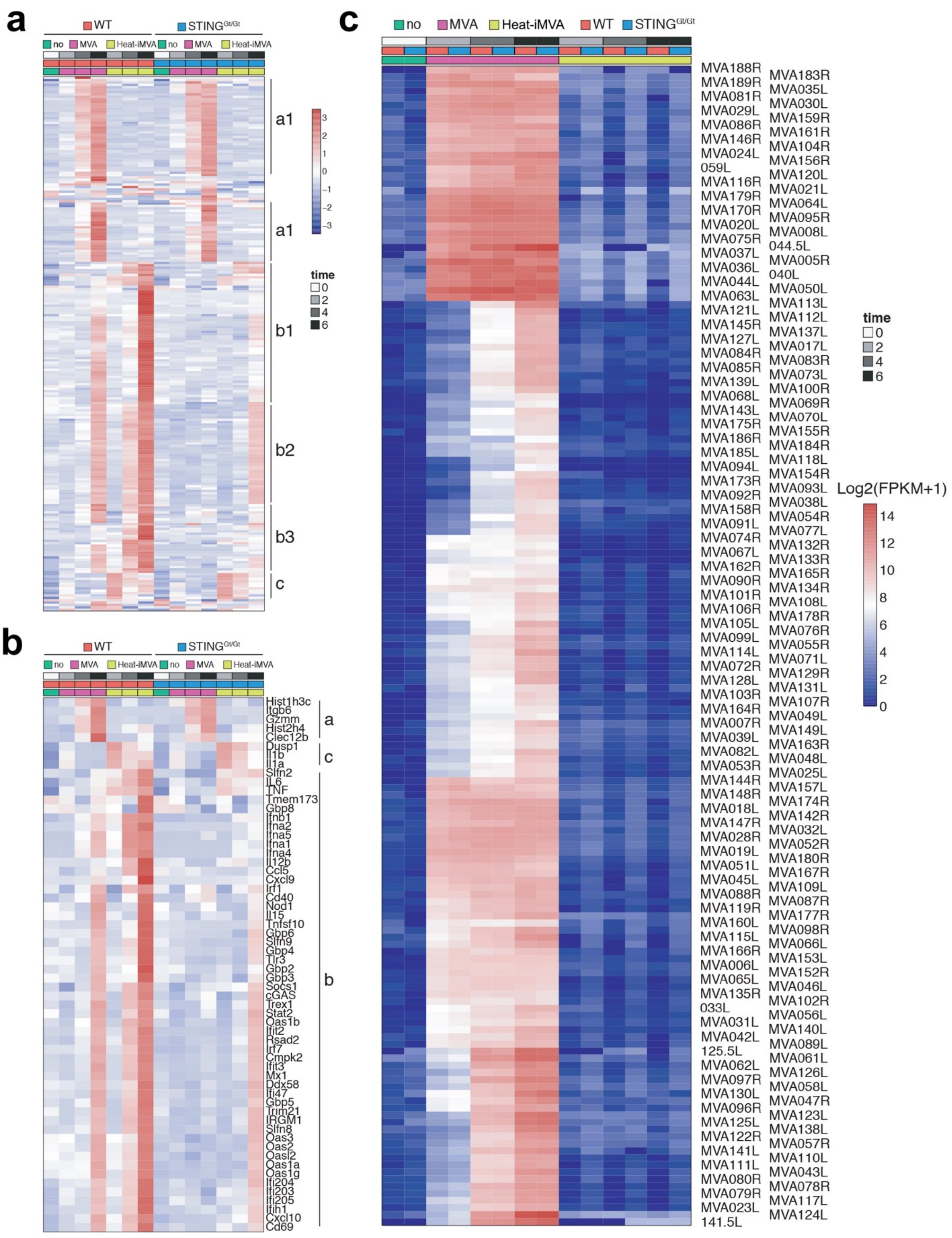

a
WT
STING^Gt/Gt
no
MVA
Heat-iMVA
no
MVA
Heat-iMVA
a1
a1
b1
b2
b3
c
3
2
1
0
−1
−2
−3
time
0
2
4
6
b
WT
STING^Gt/Gt
no
MVA
Heat-iMVA
no
MVA
Heat-iMVA
Hist1h3c
Itgb6
Gzmm
Hist2h4
Clec12b
Dusp1
Il1b
Il1a
Slfn2
Il6
TNF
Tmem173
Gbp8
Ifnb1
Ifna2
Ifna5
Ifna1
Ifna4
Il12b
Ccl5
Cxcl9
Irf1
Cd40
Nod1
Il15
Tnfsf10
Gbp6
Slfn9
Gbp4
Tlr3
Gbp2
Gbp3
Socs1
cGAS
Trex1
Stat2
Oas1b
Ifit2
Rsad2
Irf7
Cmpk2
Ifit3
Mx1
Ddx58
Ifi47
Gbp5
Trim21
IRGM1
Slfn8
Oas3
Oas2
Oasl2
Oas1a
Oas1g
Ifi204
Ifi203
Ifi205
Ifih1
Cxcl10
Cd69
a
c
b
c
no
MVA
Heat-iMVA
WT
STING^Gt/Gt
MVA188R
MVA189R
MVA081R
MVA029L
MVA086R
MVA146R
MVA024L
059L
MVA116R
MVA179R
MVA170R
MVA020L
MVA075R
MVA037L
MVA036L
MVA044L
MVA063L
MVA121L
MVA145R
MVA127L
MVA084R
MVA085R
MVA139L
MVA068L
MVA143L
MVA175R
MVA186R
MVA185L
MVA094L
MVA173R
MVA092R
MVA158R
MVA091L
MVA074R
MVA067L
MVA162R
MVA090R
MVA101R
MVA106R
MVA105L
MVA099L
MVA114L
MVA072R
MVA128L
MVA103R
MVA164R
MVA007R
MVA039L
MVA082L
MVA053R
MVA144R
MVA148R
MVA018L
MVA147R
MVA028R
MVA019L
MVA051L
MVA045L
MVA088R
MVA119R
MVA160L
MVA115L
MVA166R
MVA006L
MVA065L
MVA135R
033L
MVA031L
MVA042L
125.5L
MVA062L
MVA097R
MVA130L
MVA096R
MVA125L
MVA122R
MVA141L
MVA111L
MVA080R
MVA079R
MVA023L
141.5L
MVA183R
MVA035L
MVA030L
MVA159R
MVA161R
MVA104R
MVA156R
MVA120L
MVA021L
MVA064L
MVA095R
MVA008L
044.5L
MVA005R
040L
MVA050L
MVA113L
MVA112L
MVA137L
MVA017L
MVA083R
MVA073L
MVA100R
MVA069R
MVA070L
MVA155R
MVA184R
MVA118L
MVA154R
MVA093L
MVA038L
MVA054R
MVA077L
MVA132R
MVA133R
MVA165R
MVA134R
MVA108L
MVA178R
MVA076R
MVA055R
MVA071L
MVA129R
MVA131L
MVA107R
MVA049L
MVA149L
MVA163R
MVA048L
MVA025L
MVA157L
MVA174R
MVA142R
MVA032L
MVA052R
MVA180R
MVA167R
MVA109L
MVA087R
MVA177R
MVA098R
MVA066L
MVA153L
MVA152R
MVA046L
MVA102R
MVA056L
MVA140L
MVA089L
MVA061L
MVA126L
MVA058L
MVA047R
MVA123L
MVA138L
MVA057R
MVA110L
MVA043L
MVA078R
MVA117L
MVA124L
time
0
2
4
6
Log2(FPKM+1)
14
12
10
8
6
4
2
0

Supplementary Figure 2. Time-resolved transcriptome profiling of WT or STING$^{Gt/Gt}$ BMDCs infected with either live MVA or heat-iMVA. (**a**) A heat map of a one-way hierarchical clustering analysis of the top 200 genes ranked by Z-score of log2RPKM, indicating genes that exhibited the most statistically significant changes in gene expression over the course of the experiment. Several clusters of genes with similar gene expression changes were observed (indicated as a1-2, b1-3, and c). (**b**) A heat map of a subset of genes from panel A, showing IFN-regulated genes and genes involved in inflammation. (**c**) A heat map of a one-way hierarchical cluster analysis of MVA and Heat-iMVA transcriptome, using log2 RPKM, illustrating the temporal pattern of viral gene expression changes. Light gray: 2 h. Medium gray: 4 h. Black: 6 h.

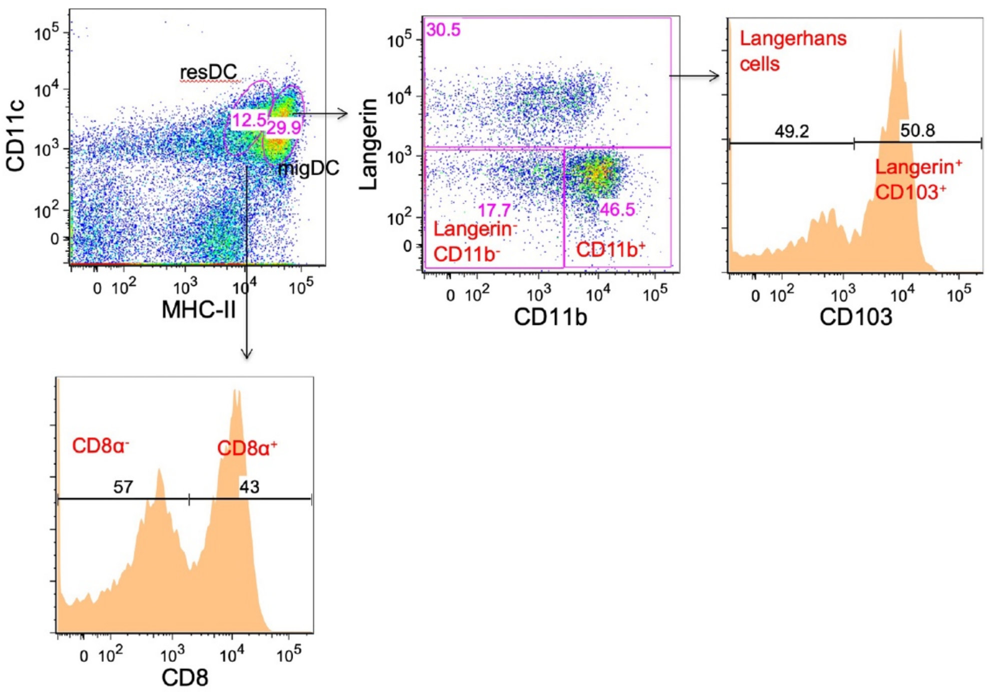

Supplementary Figure 3. Gating strategy of dendritic cll populations in skin LN. Skin LN were harvested and digested into single cell suspension. Cells were stained with a cocktail of antibodies to distinguish DC subsets. Within single cells, dead cells, CD119$^+$, DX5$^+$, TER119$^+$ and CD3ε$^+$ cells were excluded for analysis. The CD11c^{hi} MHC II^{int} population represents lymphoid resident DCs, and can be further divided into CD8$^+$ and CD8$^-$ DCs. CD11c$^{int/hi}$ MHC II^{hi} cells were further analyzed for the expression of Langerin and CD11b to define skin migratory DC subsets. Langerin$^-$ cells were divided into CD11b$^+$ DCs and CD11b$^-$ DCs. Langerin$^+$ cells were further analyzed for the expression of CD103 and divided into two groups: CD103$^+$ DCs and langerhans cells.
