# Supplementary material for: Heat-inactivated modified vaccinia virus Ankara boosts Th1 cellular and humoral immunity as a vaccine adjuvant
Source: NPJ Vaccines. 2022 Oct 19;7:120. doi: 10.1038/s41541-022-00542-5 (PMC9580433; doi:10.1038/s41541-022-00542-5)
Supplement: Supplementary file 2 — REPORTING SUMMARY [file 41541_2022_542_MOESM2_ESM.pdf]

nature portfolio

Corresponding author(s): Liang Deng

Last updated by author(s): Jul 11, 2022

Reporting Summary

Nature Portfolio wishes to improve the reproducibility of the work that we publish. This form provides structure for consistency and transparency in reporting. For further information on Nature Portfolio policies, see our Editorial Policies and the Editorial Policy Checklist.

Statistics

For all statistical analyses, confirm that the following items are present in the figure legend, table legend, main text, or Methods section.

n/a	Confirmed	
☐	☒	The exact sample size (n) for each experimental group/condition, given as a discrete number and unit of measurement
☐	☒	A statement on whether measurements were taken from distinct samples or whether the same sample was measured repeatedly
☐	☒	The statistical test(s) used AND whether they are one- or two-sided<br>*Only common tests should be described solely by name; describe more complex techniques in the Methods section.*
☐	☒	A description of all covariates tested
☐	☒	A description of any assumptions or corrections, such as tests of normality and adjustment for multiple comparisons
☐	☒	A full description of the statistical parameters including central tendency (e.g. means) or other basic estimates (e.g. regression coefficient) AND variation (e.g. standard deviation) or associated estimates of uncertainty (e.g. confidence intervals)
☐	☒	For null hypothesis testing, the test statistic (e.g. F, t, r) with confidence intervals, effect sizes, degrees of freedom and P value noted<br>*Give P values as exact values whenever suitable.*
☒	☐	For Bayesian analysis, information on the choice of priors and Markov chain Monte Carlo settings
☐	☐	For hierarchical and complex designs, identification of the appropriate level for tests and full reporting of outcomes
☒	☐	Estimates of effect sizes (e.g. Cohen's d, Pearson's r), indicating how they were calculated

Our web collection on statistics for biologists contains articles on many of the points above.

Software and code

Policy information about availability of computer code

Data collection	No software was used.
Data analysis	Flowjo 10.5.3 (Tree Star) for FACS results; GraphPad Prism 7 for statistics; Image J (1.53) for imaging data analysis.

For manuscripts utilizing custom algorithms or software that are central to the research but not yet described in published literature, software must be made available to editors and reviewers. We strongly encourage code deposition in a community repository (e.g. GitHub). See the Nature Portfolio guidelines for submitting code & software for further information.

Data

Policy information about availability of data

All manuscripts must include a data availability statement. This statement should provide the following information, where applicable:

- Accession codes, unique identifiers, or web links for publicly available datasets
- A description of any restrictions on data availability
- For clinical datasets or third party data, please ensure that the statement adheres to our policy

All data generated and supporting the findings of this study are available within this paper. RNA-sequencing data have been deposited at NCBI Short-Read Archive (SRA) and are publicly available as of the date of publication under the BioProject number PRJNA743347.

Human research participants

Policy information about studies involving human research participants and Sex and Gender in Research.

Reporting on sex and gender	*Use the terms sex (biological attribute) and gender (shaped by social and cultural circumstances) carefully in order to avoid confusing both terms. Indicate if findings apply to only one sex or gender; describe whether sex and gender were considered in study design whether sex and/or gender was determined based on self-reporting or assigned and methods used. Provide in the source data disaggregated sex and gender data where this information has been collected, and consent has been obtained for sharing of individual-level data; provide overall numbers in this Reporting Summary. Please state if this information has not been collected. Report sex- and gender-based analyses where performed, justify reasons for lack of sex- and gender-based analysis.*
Population characteristics	*Describe the covariate-relevant population characteristics of the human research participants (e.g. age, genotypic information, past and current diagnosis and treatment categories). If you filled out the behavioural & social sciences study design questions and have nothing to add here, write "See above."*
Recruitment	*Describe how participants were recruited. Outline any potential self-selection bias or other biases that may be present and how these are likely to impact results.*
Ethics oversight	*Identify the organization(s) that approved the study protocol.*

Note that full information on the approval of the study protocol must also be provided in the manuscript.

Field-specific reporting

Please select the one below that is the best fit for your research. If you are not sure, read the appropriate sections before making your selection.

☒ Life sciences ☐ Behavioural & social sciences ☐ Ecological, evolutionary & environmental sciences

For a reference copy of the document with all sections, see nature.com/documents/nr-reporting-summary-flat.pdf

Life sciences study design

All studies must disclose on these points even when the disclosure is negative.

Sample size	Sample sizes were not predetermined and are indicated in the figure legends. The group sizes of mice and samples were chosen based on our experience with similar studies, common practice in this field and resource availability.
Data exclusions	We did not exclude data in this study.
Replication	All the experimental findings were reliably reproduced as validated by at least two independent experiments.
Randomization	Samples were randomized into experimental or control groups. Animals were randomized into different treatment groups.
Blinding	The investigator for viral titer determination was blinded. Investigators were not blinded to group allocation during data collection and/or analysis in other experiments, because the same researcher performed the experiment and analyzed the data.

Reporting for specific materials, systems and methods

We require information from authors about some types of materials, experimental systems and methods used in many studies. Here, indicate whether each material, system or method listed is relevant to your study. If you are not sure if a list item applies to your research, read the appropriate section before selecting a response.

Materials & experimental systems

n/a	Involved in the study	
☐	☒	Antibodies
☐	☒	Eukaryotic cell lines
☒	☐	Palaeontology and archaeology
☐	☒	Animals and other organisms
☒	☐	Clinical data
☒	☐	Dual use research of concern

Methods

n/a	Involved in the study	
☒	☐	ChIP-seq
☐	☒	Flow cytometry
☒	☐	MRI-based neuroimaging

March 2021

Antibodies

Antibodies used	The following antibodies were used for flow cytometry: BioLegend: CD11c (N418). CD11b (M1/70), MHC-II (M5/114.15.2), CD3e (145-2C11), CD8 (53-5.8), CD4 (GK1.5), IFN-r (XMG1.2); BD Biosciences: CD19 (1D3), CD49b (DX5); Thermo Fisher: CD16/CD32 (93), CD103 (2E7), TER-119 (TER-119), CD207 (eBioL31). The following antibodies were used for ELISA: SouthernBiotech: Goat Anti-Mouse IgG1, Human ads-HRP. ThermoFisher: Goat anti-Mouse IgG2c, HRP.
Validation	The specificities of listed FACS antibodies have been validated by the manufacturer by flow cytometry.<br>CD11c (N418) Cat# 117320: https://www.biolegend.com/en-us/products/alexa-fluor-700-anti-mouse-cd11c-antibody-3429?GroupID=BLG11937<br>CD11b (M1/70) Cat# 101226: https://www.biolegend.com/en-us/products/apc-cyanine7-anti-mouse-human-cd11b-antibody-3930?GroupID=BLG10616<br>MHC II (M5/114.15.2) Cat# 107645: https://www.biolegend.com/fr-ch/search-results/brilliant-violet-785-anti-mouse-i-a-i-e-antibody-12087<br>CD3e (145-2C11) Cat# 100341: https://www.biolegend.com/en-us/products/brilliant-violet-421-anti-mouse-cd3epsilon-antibody-7132?GroupID=BLG6744<br>CD4 (GK1.5) Cat# 100428: https://www.biolegend.com/en-us/products/pacific-blue-anti-mouse-cd4-antibody-3316?GroupID=BLG4745<br>CD8 (53-5.8) Cat# 140418: https://www.biolegend.com/en-us/products/percp-cyanine5-5-anti-mouse-cd8b2-antibody-17484?GroupID=BLG8876<br>IFN-r (XMG1.2) Cat# 505810: https://www.biolegend.com/en-us/products/apc-anti-mouse-ifn-gamma-antibody-993?GroupID=GROUP24<br>CD19 (1D3) Cat# 562701: https://www.bdbiosciences.com/en-us/products/reagents/flow-cytometry-reagents/research-reagents/single-color-antibodies-ruo/bv421-rat-anti-mouse-cd19.562701<br>CD49b (DX5) Cat# 563063: https://www.bdbiosciences.com/en-us/products/reagents/flow-cytometry-reagents/research-reagents/single-color-antibodies-ruo/bv421-rat-anti-mouse-cd49b.563063<br>CD16/CD32 (93) Cat# 13-0161-82: https://www.thermofisher.com/antibody/product/CD16-CD32-Antibody-clone-93-Monoclonal/13-0161-82<br>CD207 (eBioL31) Cat# 12-2075-82: https://www.thermofisher.com/antibody/product/CD207-Langerin-Antibody-clone-eBioL31-Monoclonal/12-2075-82<br>CD103 (2E7) Cat# 11-1031-82: https://www.thermofisher.com/antibody/product/CD103-Integrin-alpha-E-Antibody-clone-2E7-Monoclonal/11-1031-82<br>TER-119 (TER-119) Cat# 48-5921-82: https://www.thermofisher.com/antibody/product/TER-119-Antibody-clone-TER-119-Monoclonal/48-5921-82<br>Goat Anti-Mouse IgG1, Human ads-HRP Cat# 1070-05: https://www.southernbiotech.com/goat-anti-mouse-igg1-human-ads-hrp-1070-05<br>Goat anti-Mouse IgG2c, HRP Cat# PA1-29288: https://www.thermofisher.com/antibody/product/Goat-anti-Mouse-IgG2c-Secondary-Antibody-Polyclonal/PA1-29288

Eukaryotic cell lines

Policy information about cell lines and Sex and Gender in Research

Cell line source(s)	BHK21 and B16-F10 cell lines were purchased from ATCC. HEK293T-hACE2 was made in our lab.
Authentication	Cell lines were not authenticated.
Mycoplasma contamination	All of the cell lines were tested negative for mycoplasma contamination.
Commonly misidentified lines (See ICLAC register)	No commonly misidentified cell lines were used.

Animals and other research organisms

Policy information about studies involving animals; ARRIVE guidelines recommended for reporting animal research, and Sex and Gender in Research

Laboratory animals	Female C57BL/6J mice between 6 and 8 weeks of age were purchased from the Jackson Laboratory and were used for vaccination experiments and for the preparation of bone marrow-derived dendritic cells. Batf3-/- mice were generated in the laboratory of Kenneth Murphy (Washington University). STINGGt/Gt mice were generated in the laboratory of Russell Vance (University of California, Berkeley). OT-1 mice were generated in the laboratory of Michael Bevan (University of Washington) and purchased from the Jackson laboratory. All mice were maintained in the animal facility at the Sloan Kettering Cancer Institute. All procedures were performed in strict accordance with the recommendations in the Guide for the Care and Use of Laboratory Animals of the National Institute of Health. The protocol was approved by the Committee on the Ethics of Animal Experiments of Sloan-Kettering Cancer Institute.
Wild animals	The study did not involve wild animals.
Reporting on sex	Female mice were used in experiments.

Field-collected samples	The study did not involve samples collected from the field.
Ethics oversight	All procedures were performed in strict accordance with the recommendations in the Guide for the Care and Use of Laboratory Animals of the National Institute of Health. The protocol was approved by the Committee on the Ethics of Animal Experiments of Sloan-Kettering Cancer Institute.

Note that full information on the approval of the study protocol must also be provided in the manuscript.

Flow Cytometry

Plots

Confirm that:

- [x] The axis labels state the marker and fluorochrome used (e.g. CD4-FITC).
- [x] The axis scales are clearly visible. Include numbers along axes only for bottom left plot of group (a 'group' is an analysis of identical markers).
- [x] All plots are contour plots with outliers or pseudocolor plots.
- [x] A numerical value for number of cells or percentage (with statistics) is provided.

Methodology

Sample preparation	To analyze antigen-specific T cells in the spleensor LNs, spleens or LNs from vaccinated mice was collected and processed using Miltenyi GentleMACS™ Dissociator. Red blood cells were lysed using ACK lysing buffer (Lonza).
Instrument	LSR Fortessa (BD Biosciences)
Software	Flowjo 10.5.3 (Tree Star)
Cell population abundance	When cells were sorted or enriched, the purity was confirmed by flow cytometry and in each case was above 90% purity.
Gating strategy	Cells were first gated by FSC/SSC. Singlets were gated according to the pattern of FSC-H vs. FSC-A. Positive populations were determined by the specific antibodies, which were distinct from negative populations.

- [x] Tick this box to confirm that a figure exemplifying the gating strategy is provided in the Supplementary Information.
